# Supplementary figures and images for: Doctor Referral of Overweight People to a Low-Energy Treatment (DROPLET) in primary care using total diet replacement products: a protocol for a randomised controlled trial
Source: BMJ Open. 2017 Aug 4;7(8):e016709. doi: 10.1136/bmjopen-2017-016709 (PMC5629710; doi:10.1136/bmjopen-2017-016709)

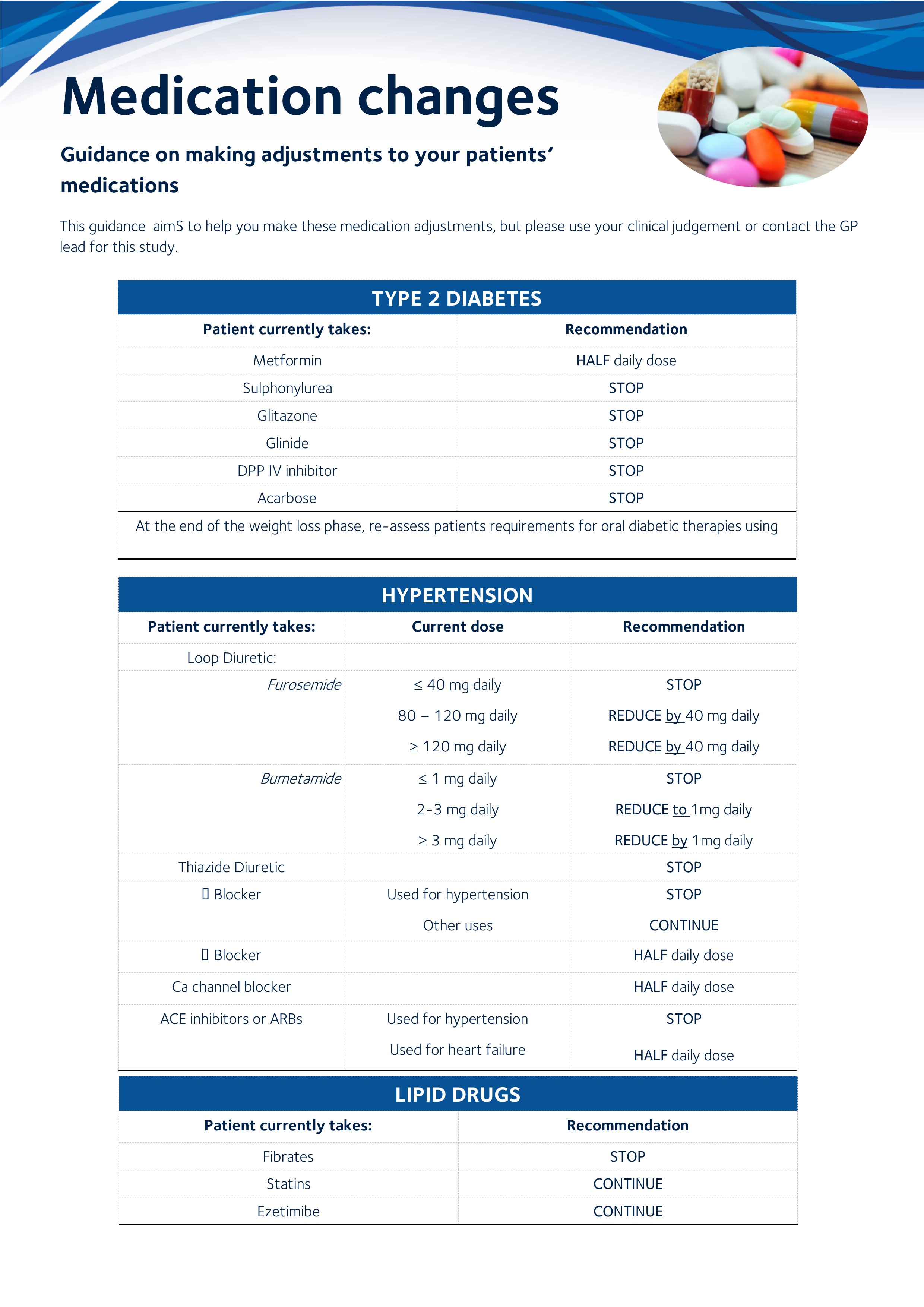

Supplement: Supplementary file 1 [file bmjopen-2017-016709supp001.jpg]
