# Supplementary material for: Doctor Referral of Overweight People to a Low-Energy Treatment (DROPLET) in primary care using total diet replacement products: a protocol for a randomised controlled trial
Source: BMJ Open. 2017 Aug 4;7(8):e016709. doi: 10.1136/bmjopen-2017-016709 (PMC5629710; doi:10.1136/bmjopen-2017-016709)
Supplement: Author's manuscript [file bmjopen-2017-016709.draft_revisions.pdf]

BMJ Open is committed to open peer review. As part of this commitment we make the peer review history of every article we publish publicly available.

When an article is published we post the peer reviewers' comments and the authors' responses online. We also post the versions of the paper that were used during peer review. These are the versions that the peer review comments apply to.

The versions of the paper that follow are the versions that were submitted during the peer review process. They are not the versions of record or the final published versions. They should not be cited or distributed as the published version of this manuscript.

BMJ Open is an open access journal and the full, final, typeset and author-corrected version of record of the manuscript is available on our site with no access controls, subscription charges or pay-per-view fees (http://bmjopen.bmj.com).

If you have any questions on BMJ Open's open peer review process please email editorial.bmjopen@bmj.com

BMJ Open

Doctor referral of overweight people to a low-energy treatment (DROPLET) in primary care using total diet replacement products: a protocol for a randomised controlled trial

Journal:	*BMJ Open*
Manuscript ID	bmjopen-2017-016709
Article Type:	Protocol
Date Submitted by the Author:	03-Mar-2017
Complete List of Authors:	Jebb, Susan; University of Oxford, Nuffield Department of Primary Care Health Sciences<br>Astbury, Nerys; University of Oxford, Nuffield Department of Primary Care Health Sciences<br>Tearne, Sarah; University of Oxford, Nuffield Department of Primary Care Health Sciences<br>Nickless, Alecia; University of Oxford, Nuffield Department of Primary Care Health Sciences<br>Aveyard, Paul; University of Oxford, Nuffield Department of Primary Care Health Sciences
**Primary Subject Heading**:	General practice / Family practice
Secondary Subject Heading:	Nutrition and metabolism
Keywords:	Obesity, Diet, Weight loss, PRIMARY CARE

SCHOLARONE™
Manuscripts

For peer review only

Doctor referral of overweight people to a low-energy treatment (DROPLET) in primary care using total diet replacement products: a protocol for a randomised controlled trial [a]

Susan A Jebb [1]
Nerys M Astbury [1]
Sarah Tearne [1]
Alecia Nickless [1]
Paul Aveyard [1]

*Corresponding Author: Nuffield Department of Primary Care Health Sciences, Radcliffe Primary Care Building, Woodstock Road, Oxford, OX2 6GG, email: nerys.astbury@phc.ox.ac.uk, tel: 01865 617871

[1] Nuffield Department of Primary Care Health Sciences, University of Oxford, Oxford UK

[a] This trial is sponsored by the University of Oxford, Clinical Trials and Research Governance, Joint Research Office, Block 60, Churchill Hospital, Old Road, Headington, Oxford, OX3 7LE, UK.

Keywords: Obesity, diet, weight loss, primary care

For peer review only

ABSTRACT

Introduction

The global prevalence of obesity has risen significantly in recent decades. There is a pressing need to identify effective interventions to treat established obesity that can be delivered at scale. The aim of the DROPLET study is to determine the clinical effectiveness, feasibility and acceptability of referral to a low-energy total diet replacement programme compared with usual weight management interventions in primary care.

Methods and Analysis

The DROPLET trial is a randomised controlled trial comparing a low-energy total diet replacement programme with usual weight management interventions delivered in primary care. Eligible patients will be recruited through primary care registers and randomised to receive a behavioural support programme delivered by their practice nurse or a referral to a commercial provider offering an initial 810 kcal/d low-energy total diet replacement programme for 8 weeks, followed by gradual food reintroduction, along with weekly behavioural support for 24 weeks. The primary outcome is weight change at 12 months. The secondary outcomes are weight change at 3 and 6 months, the proportion of participants achieving 5% and 10% weight loss at 12 months and change in fat mass, HbA1c, LDL cholesterol and systolic and diastolic blood pressure at 12 months. Data will be analysed on the basis of intention to treat. Qualitative interviews on a sub-sample of patients and healthcare providers will assess their experiences of the weight loss programmes and identify factors affecting acceptability and adherence.

Ethics and dissemination

This study has been reviewed and approved by NHS/HRA Research Ethics Committee (Ref: SC/15/0337). The trial findings will be disseminated to academic and health professionals through presentations at meetings and peer reviewed journals and to the public through the media. If the intervention is effective, the results will be communicated to policymakers and commissioners of weight management services.

Trial registration number

ISRCTN75092026

For peer review only

For peer review only - http://bmjopen.bmj.com/site/about/guidelines.xhtml

STRENGTHS & LIMITATIONS

- This study is the first randomised controlled trial of a low-energy total diet replacement programme for weight management in routine primary care and the largest randomised controlled trial to date of low-energy total diet replacement programmes for weight loss.
- This intervention is based on a model of care where GPs refer patients to a programme delivered in the community by a commercial provider, which, if successful, could be readily adopted into practice without the need for specialist training.
- The primary outcome is weight at one year. Although this is 9 months after the low-energy total diet replacement, epidemiological evidence suggests that any weight lost will continue to be regained beyond one year.
- The intention of obesity treatment programmes is to improve long-term health but this study does not include morbidity or mortality outcomes.
- The counsellors delivering the low-energy total diet replacement programme are trained to a standard protocol, whereas practice nurses delivering the comparator are not; this may introduce heterogeneity in the comparator 'usual-care' intervention.

For peer review only

INTRODUCTION

The prevalence obesity worldwide has more than doubled since 1980 [1]. According to the latest estimates from the World Health Organization (WHO) more than 1.9 billion adults were overweight, of whom 600 million were obese, representing 39% and 13% of the world's adult population, respectively [2]. Obesity is associated with premature mortality [3] but also substantial morbidity, including significantly increased risks of diabetes, cardiovascular disease and most non-smoking related cancers, as well as physical impairments linked to excess weight such as breathlessness, joint problems and back pain [4]. Collectively this creates a burden of ill-health and reduced quality of life for individuals, additional treatment costs to the NHS and reductions in economic productivity [5].

While high priority must be given to prevent future cases of obesity, in the short term there is a pressing need to identify effective interventions to treat established obesity. Research has shown that even modest reductions in weight can bring significantly reduced risks of disease. For example, in the US Diabetes Prevention Program (DPP) individuals randomised to an intensive lifestyle intervention lost 7kg by the end of the first year. Although some of this weight was regained, the intensive lifestyle group remained 4 kg lighter than the usual care group at four years and this reduced the incidence of diabetes by 58% relative to usual care [6] with benefits persisting to at least 15 year follow-up despite weight regain [7].

Primary care is an important setting for weight management interventions to reduce multi-morbidity. However, although a number of interventions have been shown to be effective in intensive research studies, this success has not always been replicated in routine settings. For example, there was no significant reduction in weight when a weight loss programme adapted from the DPP was delivered by primary care teams[8]. Our recent review of interventions suitable for use in routine care[9] and a second review, using slightly different inclusion criteria, of interventions specifically delivered in primary care [10] both concluded that behavioural weight management interventions led by primary care practitioners were ineffective. This may relate in part to the complexity of advice needed for successful dietary change and the need for frequent contact to provide support which exceeds the capacity of routine primary care systems. Currently, the most effective option for weight management in primary care is GP referral to a commercial provider offering group-based support, and our meta-analysis showed a mean reduction in weight of 2.3 kg over no intervention at one year [9]. However, greater weight losses would be expected to bring greater health gains.

Very low energy diets (VLEDs) have been used for weight loss over many years in specialist settings. A VLED is defined as a diet providing ≤800kcal a day, based on the use of specially formulated products designed as the sole source of nutrition during periods of total diet replacement. When

For Peer review only

For peer review only - http://bmjopen.bmj.com/site/about/guidelines.xhtml

used as directed, these formula products meet 100% of the dietary reference values for vitamins, minerals and trace elements and are enriched with high biological-value protein. A recent systematic review and meta-analysis of the available randomised controlled trials showed that behavioural weight management interventions incorporating a VLED led to 3.9 kg greater weight loss at one year compared with intensive specialist-delivered behavioural programmes [11]. However, most of the trials included in this review were small, typically including only 50-100 participants that were treated by obesity specialists and many trials had methodological limitations.

UK guidance from the National Institute for Health and Care Excellence (NICE) recommends that VLEDs may only be used for a maximum of 12 weeks in people who have a clinical need to lose weight rapidly, such as prior to a knee replacement surgery or those seeking fertility services, but recommends against their routine use to manage obesity [12]. Clinical guidance in the USA does not recommend the routine use of VLEDs, but rather suggests that their use "*may* be reasonable in limited circumstances, but only when provided by trained practitioners in a medical care setting where medical monitoring and high intensity lifestyle intervention can be provided" [13].

Nevertheless, there has been growing interest in the potential for routine use of weight loss programmes similar to traditional VLEDs, in so far as they incorporate a period of total diet replacement using specially formulated products as the sole source of nutrition, but where the energy content is more than 800kcal/day but less than 1600 kcal/day. The NICE guidelines suggest that this type of low-energy diet could be considered for weight management, providing care is taken to ensure they are nutritionally complete [12]. There is one observational report (n = 91) on the use of these low-energy total diet replacement programmes in primary care which found that 64% of participants completed the 810kcal/day dietary programme, defined as either 12 weeks or reaching 20kg weight loss, with a mean weight loss of 16.9 kg (standard deviation (SD) = 6.0 kg). One third of participants starting the programme maintained a weight loss of ≥15 kg at 12 months [14]. A large randomised controlled trial, the DiRECT study, is currently underway to investigate whether this type of low-energy total diet replacement programme can be used to treat type 2 diabetes among people who are also overweight [15]. It will compare the health effects of the current best-available type 2 diabetes care with those achieved through weight management based on a low-energy total diet replacement programme. While this will provide important mechanistic evidence on the links between weight loss and diabetes risk, it will be delivered by specialist staff with specific training and will not address the wider challenges associated with the use of such programmes for routine weight management programmes led by non-specialists in routine care.

To fill this evidence gap we will conduct a randomised controlled trial to specifically test the effectiveness of a GP referral to a community-based low energy total diet replacement programme

For peer review only - http://bmjopen.bmj.com/site/about/guidelines.xhtml

for patients who are obese and likely to benefit from weight loss. It will assess the clinical effectiveness of a weight loss intervention by measuring weight loss and the change in biomarkers of cardiovascular risk at 12 months relative to weight loss advice provided by practice nurses. This comparator is intended to represent 'usual care', though in practice most patients who are obese are not offered support to lose weight.

The context for this trial follows the established model for GP referral to community group-based weight loss programmes [16]. This uses the generic authority and credibility of health professionals to motivate patients to consider weight management and the specialist knowledge of the commercial provider to guide the intervention and offer frequent contact and behavioural support to the patient. If successful, it will provide another option for weight management that can be offered to patients in primary care, and GPs will be able to guide patients towards the treatment which best fits their circumstances and preferences. This trial will specifically test whether a partnership between GPs and providers will allow for the safe provision of low energy total diet replacement programmes even for patients with multi-morbidity who may gain the greatest benefits from such interventions but who may also need clinical oversight and adjustments to some of their medications as they lose weight. It will provide the opportunity for qualitative research to investigate the perspectives of patients and health care practitioners on this type of treatment.

OBJECTIVE:

The aim of the DROPLET trial is to determine the clinical effectiveness, feasibility and acceptability of referral to a low-energy total diet replacement programme compared with usual weight management interventions in primary care.

For peer review only

METHODS:

Design and Setting

The study will take place in general practices in England. Designed as an individually randomised, two arm and parallel group superiority trial with the primary endpoint as objectively measured changes in body weight from baseline to 12 months. Due to the nature of the intervention it will not be possible to blind participants, clinicians or some of the study team to the treatment allocation after randomisation.

Recruitment

Around 10 general practices will be identified to take part through the clinical research networks. Recruited practices will be asked to conduct a search of their electronic health records in order to identify suitable patients for the DROPLET study. As a result of this search, eligible patients will be sent an invitation letter from their GP as part of a staggered mail out. Patients will be encouraged to call the research team if they are interested in taking part.

GPs may also identify eligible patients during routine consultations. The GP will provide the patient with an invitation letter and suggest that the patient ring the study team. The study team will provide the potential participants with information on what taking part in the study will entail, and an initial assessment of suitability to take part. Those who make contact and self-report meeting the eligibility criteria will be scheduled for a baseline/enrolment appointment.

Inclusion Criteria:

- Participant is willing and able to give informed consent for participation in the study.
- Aged 18 years or above.
- Body Mass Index ≥30 kg/m^2.
- Likely to benefit from weight loss in the GP's opinion.

Exclusion criteria:

- Unable to understand English
- Currently or recently (within 3 months of study entry) attended a weight management programme or currently participating in another weight loss study.
- Had bariatric surgery, or scheduled bariatric surgery.
- Pregnant, breastfeeding, or planning to become pregnant during the course of the study.
- Receiving insulin therapy
- Heart attack or stroke within the last 3 months,
- Heart failure of grade II New York Heart Association and more severe
- Angina, arrhythmia, including atrial fibrillation or prolonged QT syndrome

For peer review only

- Taking MAOI medication
- Taking anticoagulant medication (e.g. warfarin)
- Taking varenicline (smoking cessation medication)
- Chronic renal failure of stage 4 or 5
- Active liver disease (except NAFLD) a past history of hepatoma or within 6 months of onset of acute hepatitis.
- People having active treatment for cancer other than skin cancer treated with curative intent by local treatment only or people taking hormonal or other long-term secondary prevention treatment after initial cancer treatment.
- Active treatment or investigation for possible or confirmed gastric or duodenal ulcer. Maintenance treatment with acid-suppression is not a contra-indication.
- Porphyria
- Scheduled for surgery within 12 months
- A member of household is already enrolled in the study
- Unwilling to provide blood samples
- Patients that the GP judges not able to meet the demands of either treatment programme or measurement schedule. This may include severe medical problems not listed above or severe psychiatric problems including substance misuse that make following the treatment programme or adhering to the protocol unlikely.

Participant Flow

The baseline/eligibility assessment will be scheduled with a practice nurse or health care assistant at their own GP practice, where informed consent for participation in the study will be obtained before eligibility will be formally assessed. After demographic information and all baseline measurements have been collected the participant will be randomised to the allocated treatment group using the online randomisation system. The patients' own GP will be notified by letter of the enrolment and randomisation of their patient, so that it may be documented on their medical record. Participants allocated to the low-energy total diet replacement programme and taking medications for type 2 diabetes, hypertension or high cholesterol will have their medications reviewed by a prescribing member of the clinical care team. During this medication review the clinician will decide what changes to medications are required at the time the participant commences the low energy total diet replacement programme, with guidance provided by the study team (Supplementary Figure 1). In addition, participants randomised to the low-energy total diet replacement group and who take anti-hypertensive medications will be provided with a home blood pressure monitor and asked to

For peer review only

record blood pressure once daily during the weight loss phase (weeks 1-12). These readings can be used to guide clinicians with any further changes in hypertension medications.

All participants will be invited to attend a 4 week follow-up appointment with the practice nurse. The main purpose of the visit is a clinical review of medication, including any adjustments required. Any changes in medication will be recorded on the concomitant medication log. Participants will be invited to attend further follow-up visits with a member of the trial team at the GP practice at 12 weeks, 6 months and 12 month following randomisation. Participant flow through the study is outlined in **Figure 1**.

Sample size

The total number of participants to be recruited for this study is 270. This is based on a sample size calculation for the primary outcome using equal variance independent samples t-test assuming a difference between groups at 12 months of 4kg with a standard deviation in both groups of 9kg; obtained from a meta-analysis of published studies [11]. The sample size has been inflated by 20%, to account for attrition, and assumes 90% power and two-sided alpha of 5%.

Randomisation

All eligible, consenting participants will be randomised with an allocation ratio of 1:1 to low-energy total diet replacement or usual care programmes using an online program which reveals group allocation as per a computer-generated randomisation list. The randomisation criteria will be validated by an independent statistician. Allocation will be stratified by GP Practice and baseline BMI (≤ 35kg/m2 or > $35kg/m^2$) using stratified block randomisation with randomly varying block sizes of size 2, 4, and 6. The randomisation software ensures full allocation concealment, with the allocation group only revealed to the person performing the randomisation once a study identifier and required stratification details have been entered.

For peer review only - http://bmjopen.bmj.com/site/about/guidelines.xhtml

Interventions

Low-energy total diet replacement

The programme offered to participants randomised to the active intervention will be provided by Cambridge Weight Plan Ltd.™ Northants., United Kingdom.

Following randomisation participants allocated to this group will be referred to a local Cambridge Weight Plan Counsellor who will invite the participant to attend regular appointments for 24 weeks. These appointments consist of motivational support, encouragement, reassurance and problem-solving.

During the first 12 weeks the participant will meet with their counsellor weekly. Patients will be asked to follow a programme based on using formula meal replacement products (soups, shakes and bars) and milk comprising 810kcal/day (3389kJ/d). For the first eight weeks patients will be advised to replace *all* their usual foods and drinks with four of the formula products daily, 750ml of skimmed milk, 2.25L of water or other non-calorific drinks and a fibre supplement (total diet replacement stage). During the first two weeks, the formula products will be limited to liquid products (soups and shakes), but from week 3 onwards participants will have the option to include meal replacement bars as part of the formula product allowance. After eight weeks there will be a four week stepwise reduction in the use of formula meal replacement products and a gradual re-introduction of food-based meals. The weight maintenance phase from week 12 to 24 will involve consuming only one formula product a day, with the remainder of diet provided by food. This weight maintenance phase will include a recommendation to return to the total diet replacement stage for periods of up to 4 weeks if a participant regains 1kg or more than their weight measured at 12 weeks.

All consultations with consultants and formula products will be provided to participants by their nominated consultant and will be free of charge for the first 24 weeks, after which the intervention will end. Participants in both groups will be free to choose whether or not to continue with the programme, but at their own cost.

Comparator

The comparator intervention will consist of the usual weight management programme provided by a member of the practice nurse team who has been trained to offer a weight loss programme. The trial will take place only in practices where this is routine care. Participants allocated to the usual care group will not be prevented from attending other weight management groups if they choose to do so, but no NHS referrals to these schemes will be offered during the trial. The practice nurse will give participants a copy of the booklet " So you want to lose weight … for good" [17]. This 47 page booklet provides advice akin to a behavioural weight management programme. The aim is to

For peer review only

produce a weight loss goal of 0.5 to 1kg/week. It includes goal setting, advice on portion control and physical activity, other behavioural strategies, and monitoring and feedback on progress. Nurses will be asked to offer a programme for 12 weeks, at a frequency that is usually used in the practice (e.g. weekly or bi-weekly).

Outcomes:

Primary outcome

- Change in body weight from baseline to 12 months

Secondary outcomes

- Change in body weight from baseline at 3 and 6 months
- Proportion of participants achieving 5% and 10% weight loss at 12 months
- Change in fat mass between baseline and 12 months
- Change in LDL cholesterol concentrations between baseline and 12 months
- Change in HbA1c between baseline and 12 months
- Change in systolic and diastolic blood pressure between baseline and 12 months

Exploratory outcomes

- Change in fat mass from baseline to 12 weeks and from baseline to 6 months.
- Change in waist circumference from baseline to 3, 6, and 12 months.
- Change in triglyceride and HDL cholesterol concentrations between baseline and 12 months.
- Change in fasting glucose and insulin concentrations and change in HOMA-IR, HOMA-%S and HOMA-%B between baseline and 12 months.
- Change in systolic and diastolic blood pressure between baseline and 3 months and between baseline and 6 months.
- Change in QRISK between baseline and 12 months.
- Change in the EQ-5D scale between baseline and 12 months
- Change in obesity related quality of life measured with the OWLQOL between baseline, 3, 6, and 12 months.
- Proportion of people continuing their weight loss attempt and following the prescribed programme at 4, 8, and 12 weeks.
- The number of weight control behaviours that participants are using assessed using the OxFAB questionnaire [18] at 3 and 6 months.
- Qualitative interviews with a sub-sample of participants at 6 and 12 months
- Adverse Event reports up to 12 weeks, the end of the weight loss intervention or 6 months for AE's known or presumed to be related to gallstones.

For peer review only

Measurements

Figure 2 provides a summary of the measurements collected.

Socio-demographic characteristics; Participants' will be asked to self-report age, sex, ethnicity

Medical History; Relevant medical history and all concomitant medication will be recorded and checked against the participants' medical record. Participants will also be asked to self-report items required to determine cardiovascular risk score using QRISK2 [19].

Physical measurements; Height will be measured to the nearest 1cm using stadiometers available in the practice. Weight will be recorded to the nearest 0.1kg using an electronic scale (SC240 MA, Tanita Japan) which will also record the proportion of body fat using bioelectrical impedance. Waist circumference will be measured in the horizontal plane at the upper border of the iliac crest at the end of expiration[20] using a fibreglass non-stretch tape measure fitted with a tensioning device (Gulik II Tape Meaure, Fitness Mart USA). Seated blood pressure will be measured in triplicate with 1 min between each measure. All physical measures are performed by assessors trained according to the study manual of procedures.

Fasting blood sample; A fasting venous blood sample will be collected (to be analysed for glucose, insulin, HbA1c, HDL and LDL cholesterol, triglycerides). When baseline/enrolment appointments are scheduled at times when it may be inappropriate to fast, participants will be asked to arrange for a fasting blood sample to be collected at an alternative appointment within 7 days of the enrolment visit and before the participant commences the allocated weight loss programme.

Questionnaires; Participants will be provided with a questionnaire booklet which they will be asked to complete and return to the trial team in a postage paid envelope provided. The questionnaire booklet contains the following measures:

Obesity specific quality of life (OWLQOL); a weight-specific instrument intended to be used to assess obesity specific symptoms and quality of life, general functional status and well-being, and person-specific preference measurement [21].

Quality of Life; EQ-5D will be used as a standardised validated instrument used for measuring general health status [22].

Programme adherence; Self-reported adherence to the allocated programme and methods participants are using to attempt to lose weight will be recorded by questionnaire.

Programme feedback; will be assessed using several 5-point Likert scales, including whether there is an aim to continue with the programme.

Oxford Food and Activity Behaviours (OxFAB): a questionnaire to assess personal strategies used by individuals for the purposes of weight loss[18].

Retention and withdrawal

We will seek to follow up all participants except those who expressly withdraw from the study. Participants who decide to withdraw from or discontinue the intervention allocated as part of the study will be asked to return for follow-up visits to collect outcome measures. To promote participant retention and complete follow-up participants will be offered a £10 gift card for attending each of the 6 month and 12 month follow-up visits.

Adverse Events

Adverse events are of relevance in this trial because many practitioners feel these programmes are poorly tolerated and unsuitable for routine use in primary care. We will record AEs following Good Clinical Practice (GCP). All serious and non-serious AE's that occur during the first 12 weeks of the study or until the termination of the weight loss programme will be recorded in participants who initiate one of the weight loss interventions. We will also record all AEs that are presumed to be or known to be related to gallstones up to 6 months.

Data management

Data will be recorded in a web-based data capture system (OpenClinica), which is hosted by the Primary Care Clinical Trials Unit of the University Oxford. This system is customised and has an audit trail facility. Ranges and programmed validation checks are implemented in the system in order to aid reliable data entry.

Statistical Analysis

The primary and secondary outcomes will be assessed using an intention-to-treat (ITT) analysis by an independent statistician. Each continuous outcome will be assumed to follow the normal distribution and be analysed by means of a linear mixed effects model, adjusted for outcome at baseline. The model will include fixed effects terms for randomised group, visit, interaction between randomised group and visit, and baseline BMI (for non-weight outcomes only), and random effects to account for repeated measures on the same participant at 3, 6 and 12 months. No adjustment will be made for baseline BMI in the analysis of the weight outcomes due to its strong collinearity with baseline weight. A random effect will also be included for individual practice. An unstructured variance covariance matrix will be specified between repeated measurements on the same individual and the random effects for patient and practice will be assumed to be independent. The adjusted treatment effect together with the 95% confidence interval and p-value will be reported. The analysis will be performed using PROC MIXED in a current version of SAS. The proportion of

For peer review only - http://bmjopen.bmj.com/site/about/guidelines.xhtml

participants who lose 5% and 10% of their initial weight at 12 months respectively will be presented and the adjusted difference between two arms and 95% confidence interval will be reported. The binary outcome will be analysed by means of a logistic mixed effects model, adjusting for baseline BMI (fixed effect) and practice (random effect). The number needed to treat (NNT) to achieve 5 or 10% weight loss, defined as the inverse of the absolute difference in proportions, will be reported if the differences between the treatment and control groups are statistically significant. A full statistical analysis plan will be prepared prior to any data analysis.

Qualitative sub-study

The purpose of this study is to examine participants' views of the programmes. In particular, we aim to examine the features that helped or hindered adherence to the programme and participants' views of the behavioural support provided in the respective programmes. We will therefore purposively sample participants based on their responses to the satisfaction questionnaire, reflecting positive, neutral and negative evaluations. Where possible, we will select participants to reflect both genders, socioeconomic status, and ethnic group differences. We anticipate interviewing around 20 participants in the intervention group and 10 in the control group but sampling will continue until saturation is reached, evidenced by no new themes occurring.

We will develop a semi-structured topic guide for the interviews. The interviewer will encourage respondents to discuss their perceptions and experiences freely and in depth. The interview will set the context by asking about previous experience of weight management. Thereafter, we will ask for participants' views on which component parts of their treatment they felt were effective and which they felt were not effective; thoughts about ability to continue to manage their weight when treatment has ended; and their views on medication adjustments where these occurred. The acceptability of the weight management treatment programmes and any preference they initially had for the total diet replacement programme or the usual care programme will be explored.

Data from participants will be collected in a confidential, telephone interview which will be audio recorded. All interviews will be transcribed. To examine saturation, analysis will proceed concurrently with interviewing.

Trial oversight

An independent Trial Steering Committee (TSC) will provide oversight of all matters relating to participant safety and data quality and value to the public. Due to the low risk nature of the DROPLET trial and that it is an open label trial, the TSC also has the role of the Data Monitoring

For peer review only - http://bmjopen.bmj.com/site/about/guidelines.xhtml

Committee in addition to their role as the TSC. However, there are no early stopping rules and all AEs are evaluated un-blinded to allocation by the trial management group as well as the TSC. The TSC includes an independent clinician, dietitian, statistician and two patient representatives. The TSC has reviewed the trial protocol, statistical analysis plan and the suitability of the proposed safety data to be collected. No interim analysis is planned for this trial due to the short recruitment period and low risk nature of the two dietary approaches [11]. The trial may be subject to inspection and audit by University of Oxford, under their remit as sponsor, the trial coordinating centre as the Sponsor's delegate and other regulatory bodies.

Ethics and dissemination

The study protocol (Version: 4.0 Date: 5th October 2016) was reviewed and approved by the South Central Oxford B REC Committee (Ref: 157/SC/0337). Any protocol modifications will be sent for review by the research ethics committee and will be amended at the trial registry.

It is planned that results will be disseminated to academic and health professional audiences via presentations at conferences and publication in peer-reviewed journals. Participants will be sent a summary of the trial findings at the time when the main article is published. If the trial shows this intervention is effective, results will be communicated to policymakers and commissioners of weight management services through briefing papers summarising the main findings. We will also provide the results to all participants coincident with publication and disseminate the results to the public through a press release, regardless of what the results show.

Acknowledgements

The low-energy total diet replacement programme including the formula meal replacement products will be provided by Cambridge Weight Plan Ltd, Northants, UK.

Funding and Sponsorship

This research is funded by research grants from Cambridge Weight Plan Ltd and NIHR Collaboration for Leadership in Applied Health Research and Care (CLAHRC) Oxford at Oxford Health NHS Foundation to the University of Oxford. The sponsor of the trial is the University of Oxford.

Contributions

The protocol was initiated and designed by the investigators who have no personal financial relationships with the Cambridge Weight Plan Ltd. Although Cambridge Weight Plan were consulted and commented on the protocol, the final decisions lay with the investigators. There are no

For peer review only

restrictions on publication of results arising from this study and the contract between the funder and the University ensures that the funding body will have no input into the decisions regarding publication.

SAJ and PA designed the study and secured the funding. NA and ST helped to develop the protocol. NA is the trial manger and AN is the trial statistician.

Competing interests

SAJ and PA have led publicly funded trials in which the weight management intervention was provided free of charge by other commercial companies. They receive no personal financial benefits from these trials or from the companies. NMA, ST, and AN have no competing interests.

Footnotes

As sponsor, the University of Oxford has a specialist insurance policy in place that would operate in the event of any participant suffering harm as a result of their involvement in the research.

Provenance and peer review

Not commissioned; external peer review for ethical approval prior to submission.

Data sharing

For access to the data set, a formal request should be sent to the DROPLET study group. The request will only be considered when the principal results of the study have been published.

For peer review only

References

1. Kelly T, Yang W, Chen C-S, et al. Global burden of obesity in 2005 and projections to 2030. International journal of obesity 2008;**32**(9):1431-37.
2. World Health Organization. Obesity and overweight: fact sheet Geneva: World Health Organization; 2016 [Available from: http://www.who.int/mediacentre/factsheets/fs311/en/.
3. Prospective Studies C, Whitlock G, Lewington S, et al. Body-mass index and cause-specific mortality in 900 000 adults: collaborative analyses of 57 prospective studies. Lancet 2009;**373**(9669):1083-96 doi: 10.1016/S0140-6736(09)60318-4.
4. Guh DP, Zhang W, Bansback N, et al. The incidence of co-morbidities related to obesity and overweight: a systematic review and meta-analysis. BMC Public Health 2009;**9**:88 doi: 10.1186/1471-2458-9-88.
5. Butland B, Jebb SA, McPherson K, et al. Tackling Obesities: Foresight report: Government Office for Science, 2007.
6. Knowler WC, Barrett-Connor E, Fowler SE, et al. Reduction in the incidence of type 2 diabetes with lifestyle intervention or metformin. N Engl J Med 2002;**346**(6):393-403 doi: 10.1056/NEJMoa012512.
7. Diabetes Prevention Program Research G. Long-term effects of lifestyle intervention or metformin on diabetes development and microvascular complications over 15-year follow-up: the Diabetes Prevention Program Outcomes Study. Lancet Diabetes Endocrinol 2015;**3**(11):866-75 doi: 10.1016/S2213-8587(15)00291-0.
8. Vermunt PW, Milder IE, Wielaard F, et al. Lifestyle counseling for type 2 diabetes risk reduction in Dutch primary care: results of the APHRODITE study after 0.5 and 1.5 years. Diabetes Care 2011;**34**(9):1919-25 doi: 10.2337/dc10-2293.
9. Hartmann-Boyce J, Johns DJ, Jebb SA, et al. Behavioural weight management programmes for adults assessed by trials conducted in everyday contexts: systematic review and meta-analysis. Obes Rev 2014;**15**(11):920-32 doi: 10.1111/obr.12220.
10. Booth HP, Prevost TA, Wright AJ, et al. Effectiveness of behavioural weight loss interventions delivered in a primary care setting: a systematic review and meta-analysis. Fam Pract 2014;**31**(6):643-53 doi: 10.1093/fampra/cmu064.
11. Parretti HM, Jebb SA, Johns DJ, et al. Clinical effectiveness of very-low-energy diets in the management of weight loss: a systematic review and meta-analysis of randomized controlled trials. Obes Rev 2016;**17**(3):225-34 doi: 10.1111/obr.12366.
12. (NICE) National Institute for Health and Clinical Excellence Obesity: identification, assesment and management (NICE clinical guideline). London: NICE, 2006.
13. Jensen MD, Ryan DH, Apovian CM, et al. 2013 AHA/ACC/TOS guideline for the management of overweight and obesity in adults: a report of the American College of Cardiology/American Heart Association Task Force on Practice Guidelines and The Obesity Society. Circulation 2014;**129**(25 Suppl 2):S102-38 doi: 10.1161/01.cir.0000437739.71477.ee.
14. Lean M, Brosnahan N, McLoone P, et al. Feasibility and indicative results from a 12-month low-energy liquid diet treatment and maintenance programme for severe obesity. Br J Gen Pract 2013;**63**(607):e115-24 doi: 10.3399/bjgp13X663073.
15. Leslie WS, Ford I, Sattar N, et al. The Diabetes Remission Clinical Trial (DiRECT): protocol for a cluster randomised trial. BMC Fam Pract 2016;**17**:20 doi: 10.1186/s12875-016-0406-2.
16. Ahern AL, Olson AD, Aston LM, et al. Weight Watchers on prescription: an observational study of weight change among adults referred to Weight Watchers by the NHS. BMC Public Health 2011;**11**:434 doi: 10.1186/1471-2458-11-434.
17. British Heart Foundation. *So you want to lose weight... for good*. London: BHF Publications, 2005.
18. Hartmann-Boyce J, Aveyard P, Koshiaris C, et al. Development of tools to study personal weight control strategies: OxFAB taxonomy. Obesity (Silver Spring) 2016;**24**(2):314-20 doi: 10.1002/oby.21341.

For peer review only - http://bmjopen.bmj.com/site/about/guidelines.xhtml

19. Hippisley-Cox J, Coupland C, Vinogradova Y, et al. Predicting cardiovascular risk in England and Wales: prospective derivation and validation of QRISK2. BMJ 2008;**336**(7659):1475-82 doi: 10.1136/bmj.39609.449676.25.
20. NHLBI Obesity Education Initiative. The practical guide: Identification, evaluation and treatment of overweight and obesity in adults: National Institutes of Health (NIH Publication Number 00-4084), 2000.
21. Patrick DL, Bushnell DM, Rothman M. Performance of two self-report measures for evaluating obesity and weight loss. Obes Res 2004;**12**(1):48-57 doi: 10.1038/oby.2004.8.
22. EuroQol G. EuroQol--a new facility for the measurement of health-related quality of life. Health Policy 1990;**16**(3):199-208.

For peer review only

Figure 1: Participant flow through the study

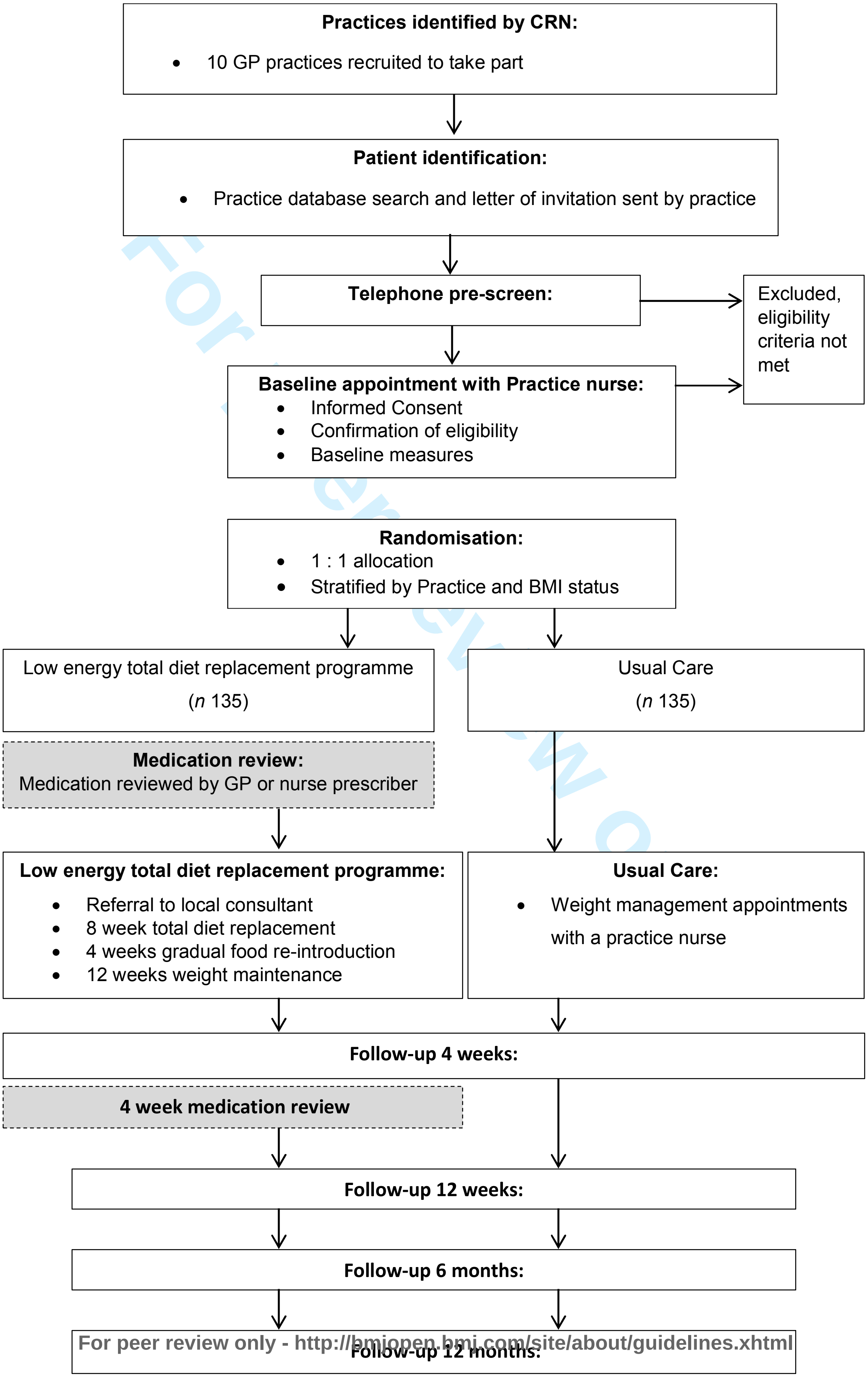

Figure 2: Schedule of measurements

	VISIT				
	Enrolment	Follow-up visits			
**TIMEPOINT**	***Baseline***	***4 weeks***	***12 weeks***	***6 months***	***12 months***
**ENROLMENT:**					
**Informed consent**	X				
**Eligibility screen**	X				
**Randomisation**	X				
**INTERVENTIONS:**					
Low energy total diet replacement programme		→	→	→	
Usual Care		→	→		
**ASSESSMENTS:**					
Demographic	X				
Medical History	X				
Concomitant Medication	X	X	X	X	X
Height	X				
Weight	X	X	X	X	X
Body composition	X	X	X	X	X
Waist circumference	X	X	X	X	X
Blood Pressure	X	X	X	X	X
Fasting blood sample	X				X
Medication review	X	X			
**QUALITATIVE INTERVIEWS**			X		X
**QUESTIONNAIRES:**					
Quality of life: (EQ-5D and OWLQOL)	X		X	X	X
Programme adherence		X	X	X	X
Programme feedback		X	X	X	
OXFAB[1]			X	X	

For peer review only

Figure 2: Schedule of measurements

1. Hartmann-Boyce J, Aveyard P, Koshiaris C, et al. Development of tools to study personal weight control strategies: OxFAB taxonomy. Obesity (Silver Spring) 2016;**24**(2):314-20 doi: 10.1002/oby.21341.

For peer review only

Medication changes

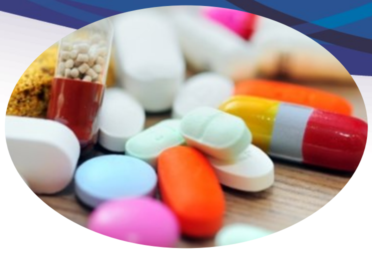

Guidance on making adjustments to your patients' medications

This guidance aims to help you make medication adjustments, but if in doubt please use your clinical judgement or contact the GP lead for this study.

The adjustments detailed below should be made when commencing the low-energy total diet replacement programme.

TYPE 2 DIABETES

Patient currently takes:	Recommendation
Metformin	HALF daily dose
Sulphonylurea	STOP
Glitazone	STOP
Glinide	STOP
DPP IV inhibitor	STOP
Acarbose	STOP

At the end of the weight loss phase, re-assess patients requirements for oral diabetic therapies using

HYPERTENSION

Patient currently takes:		Current dose	Recommendation
Loop Diuretic:			
	Furosemide	≤ 40 mg daily	STOP
		80 – 120 mg daily	REDUCE by 40 mg daily
		≥ 120 mg daily	REDUCE by 40 mg daily
	Bumetamide	≤ 1 mg daily	STOP
		2-3 mg daily	REDUCE to 1mg daily
		≥ 3 mg daily	REDUCE by 1mg daily
Thiazide Diuretic			STOP
β Blocker		Used for hypertension	STOP
		Other uses	CONTINUE
α Blocker			HALF daily dose
Ca channel blocker			HALF daily dose
ACE inhibitors or ARBs		Used for hypertension	STOP
		Used for heart failure	HALF daily dose

LIPID DRUGS

Patient currently takes:	Recommendation
Fibrates	STOP
Statins	CONTINUE
Ezetimibe	CONTINUE

For peer review only

STANDARD PROTOCOL ITEMS: RECOMMENDATIONS FOR INTERVENTIONAL TRIALS

SPIRIT 2013 Checklist: Recommended items to address in a clinical trial protocol and related documents*

Section/item	Item No	Description	Addressed on page number
**Administrative information**			
Title	1	Descriptive title identifying the study design, population, interventions, and, if applicable, trial acronym	P 1
Trial registration	2a	Trial identifier and registry name. If not yet registered, name of intended registry	P2
	2b	All items from the World Health Organization Trial Registration Data Set	
Protocol version	3	Date and version identifier	P15
Funding	4	Sources and types of financial, material, and other support	P15
Roles and responsibilities	5a	Names, affiliations, and roles of protocol contributors	P1
	5b	Name and contact information for the trial sponsor	P1
	5c	Role of study sponsor and funders, if any, in study design; collection, management, analysis, and interpretation of data; writing of the report; and the decision to submit the report for publication, including whether they will have ultimate authority over any of these activities	P15

For peer review only - http://bmjopen.bmj.com/site/about/guidelines.xhtml

	5d	Composition, roles, and responsibilities of the coordinating centre, steering committee, endpoint adjudication committee, data management team, and other individuals or groups overseeing the trial, if applicable (see Item 21a for data monitoring committee)	P14
**Introduction**			
Background and rationale	6a	Description of research question and justification for undertaking the trial, including summary of relevant studies (published and unpublished) examining benefits and harms for each intervention	P4-6
	6b	Explanation for choice of comparators	P10-11
Objectives	7	Specific objectives or hypotheses	P6
Trial design	8	Description of trial design including type of trial (eg, parallel group, crossover, factorial, single group), allocation ratio, and framework (eg, superiority, equivalence, noninferiority, exploratory)	P7
**Methods: Participants, interventions, and outcomes**			
Study setting	9	Description of study settings (eg, community clinic, academic hospital) and list of countries where data will be collected. Reference to where list of study sites can be obtained	P7
Eligibility criteria	10	Inclusion and exclusion criteria for participants. If applicable, eligibility criteria for study centres and individuals who will perform the interventions (eg, surgeons, psychotherapists)	P7-8
Interventions	11a	Interventions for each group with sufficient detail to allow replication, including how and when they will be administered	P10-11
	11b	Criteria for discontinuing or modifying allocated interventions for a given trial participant (eg, drug dose change in response to harms, participant request, or improving/worsening disease)	P13
	11c	Strategies to improve adherence to intervention protocols, and any procedures for monitoring adherence (eg, drug tablet return, laboratory tests)	P13
	11d	Relevant concomitant care and interventions that are permitted or prohibited during the trial	N/A

For peer review only - http://bmjopen.bmj.com/site/about/guidelines.xhtml

Outcomes	12	Primary, secondary, and other outcomes, including the specific measurement variable (eg, systolic blood pressure), analysis metric (eg, change from baseline, final value, time to event), method of aggregation (eg, median, proportion), and time point for each outcome. Explanation of the clinical relevance of chosen efficacy and harm outcomes is strongly recommended	P11
Participant timeline	13	Time schedule of enrolment, interventions (including any run-ins and washouts), assessments, and visits for participants. A schematic diagram is highly recommended (see Figure)	Figure 2
Sample size	14	Estimated number of participants needed to achieve study objectives and how it was determined, including clinical and statistical assumptions supporting any sample size calculations	P9
Recruitment	15	Strategies for achieving adequate participant enrolment to reach target sample size	P7
**Methods: Assignment of interventions (for controlled trials)**			
Allocation:			
Sequence generation	16a	Method of generating the allocation sequence (eg, computer-generated random numbers), and list of any factors for stratification. To reduce predictability of a random sequence, details of any planned restriction (eg, blocking) should be provided in a separate document that is unavailable to those who enrol participants or assign interventions	P9
Allocation concealment mechanism	16b	Mechanism of implementing the allocation sequence (eg, central telephone; sequentially numbered, opaque, sealed envelopes), describing any steps to conceal the sequence until interventions are assigned	P9
Implementation	16c	Who will generate the allocation sequence, who will enrol participants, and who will assign participants to interventions	P9
Blinding (masking)	17a	Who will be blinded after assignment to interventions (eg, trial participants, care providers, outcome assessors, data analysts), and how	P9
	17b	If blinded, circumstances under which unblinding is permissible, and procedure for revealing a participant's allocated intervention during the trial	N/A
**Methods: Data collection, management, and analysis**			

For peer review only - http://bmjopen.bmj.com/site/about/guidelines.xhtml

Data collection methods	18a	Plans for assessment and collection of outcome, baseline, and other trial data, including any related processes to promote data quality (eg, duplicate measurements, training of assessors) and a description of study instruments (eg, questionnaires, laboratory tests) along with their reliability and validity, if known. Reference to where data collection forms can be found, if not in the protocol	P12
	18b	Plans to promote participant retention and complete follow-up, including list of any outcome data to be collected for participants who discontinue or deviate from intervention protocols	P13
Data management	19	Plans for data entry, coding, security, and storage, including any related processes to promote data quality (eg, double data entry; range checks for data values). Reference to where details of data management procedures can be found, if not in the protocol	P13
Statistical methods	20a	Statistical methods for analysing primary and secondary outcomes. Reference to where other details of the statistical analysis plan can be found, if not in the protocol	P13/14
	20b	Methods for any additional analyses (eg, subgroup and adjusted analyses)	P13/14
	20c	Definition of analysis population relating to protocol non-adherence (eg, as randomised analysis), and any statistical methods to handle missing data (eg, multiple imputation)	P13
**Methods: Monitoring**			
Data monitoring	21a	Composition of data monitoring committee (DMC); summary of its role and reporting structure; statement of whether it is independent from the sponsor and competing interests; and reference to where further details about its charter can be found, if not in the protocol. Alternatively, an explanation of why a DMC is not needed	P14/15
	21b	Description of any interim analyses and stopping guidelines, including who will have access to these interim results and make the final decision to terminate the trial	N/A
Harms	22	Plans for collecting, assessing, reporting, and managing solicited and spontaneously reported adverse events and other unintended effects of trial interventions or trial conduct	P13
Auditing	23	Frequency and procedures for auditing trial conduct, if any, and whether the process will be independent from investigators and the sponsor	P15

For peer review only - http://bmjopen.bmj.com/site/about/guidelines.xhtml

**Ethics and dissemination**			
Research ethics approval	24	Plans for seeking research ethics committee/institutional review board (REC/IRB) approval	P15
Protocol amendments	25	Plans for communicating important protocol modifications (eg, changes to eligibility criteria, outcomes, analyses) to relevant parties (eg, investigators, REC/IRBs, trial participants, trial registries, journals, regulators)	P15
Consent or assent	26a	Who will obtain informed consent or assent from potential trial participants or authorised surrogates, and how (see Item 32)	P8
	26b	Additional consent provisions for collection and use of participant data and biological specimens in ancillary studies, if applicable	P8
Confidentiality	27	How personal information about potential and enrolled participants will be collected, shared, and maintained in order to protect confidentiality before, during, and after the trial	_____________
Declaration of interests	28	Financial and other competing interests for principal investigators for the overall trial and each study site	P16
Access to data	29	Statement of who will have access to the final trial dataset, and disclosure of contractual agreements that limit such access for investigators	_P16
Ancillary and post-trial care	30	Provisions, if any, for ancillary and post-trial care, and for compensation to those who suffer harm from trial participation	_N/A
Dissemination policy	31a	Plans for investigators and sponsor to communicate trial results to participants, healthcare professionals, the public, and other relevant groups (eg, via publication, reporting in results databases, or other data sharing arrangements), including any publication restrictions	P15
	31b	Authorship eligibility guidelines and any intended use of professional writers	P15-16
	31c	Plans, if any, for granting public access to the full protocol, participant-level dataset, and statistical code	P16
**Appendices**			

For peer review only - http://bmjopen.bmj.com/site/about/guidelines.xhtml

Informed consent materials	32	Model consent form and other related documentation given to participants and authorised surrogates	______________
Biological specimens	33	Plans for collection, laboratory evaluation, and storage of biological specimens for genetic or molecular analysis in the current trial and for future use in ancillary studies, if applicable	______________

*It is strongly recommended that this checklist be read in conjunction with the SPIRIT 2013 Explanation & Elaboration for important clarification on the items. Amendments to the protocol should be tracked and dated. The SPIRIT checklist is copyrighted by the SPIRIT Group under the Creative Commons "Attribution-NonCommercial-NoDerivs 3.0 Unported" license.

For peer review only

BMJ Open

Doctor referral of overweight people to a low-energy treatment (DROPLET) in primary care using total diet replacement products: a protocol for a randomised controlled trial

Journal:	*BMJ Open*
Manuscript ID	bmjopen-2017-016709.R1
Article Type:	Protocol
Date Submitted by the Author:	08-Jun-2017
Complete List of Authors:	Jebb, Susan; University of Oxford, Nuffield Department of Primary Care Health Sciences<br>Astbury, Nerys; University of Oxford, Nuffield Department of Primary Care Health Sciences<br>Tearne, Sarah; University of Oxford, Nuffield Department of Primary Care Health Sciences<br>Nickless, Alecia; University of Oxford, Nuffield Department of Primary Care Health Sciences<br>Aveyard, Paul; University of Oxford, Nuffield Department of Primary Care Health Sciences
**Primary Subject Heading**:	General practice / Family practice
Secondary Subject Heading:	Nutrition and metabolism
Keywords:	Obesity, Diet, Weight loss, PRIMARY CARE

SCHOLARONE™ Manuscripts

For peer review only

Doctor referral of overweight people to a low-energy treatment (DROPLET) in primary care using total diet replacement products: a protocol for a randomised controlled trial [a]

Susan A Jebb [1]

Nerys M Astbury [1]

Sarah Tearne [1]

Alecia Nickless [1]

Paul Aveyard [1]

*Corresponding Author: Nuffield Department of Primary Care Health Sciences, Radcliffe Primary Care Building, Woodstock Road, Oxford, OX2 6GG, email: nerys.astbury@phc.ox.ac.uk, tel: 01865 617871

[1] Nuffield Department of Primary Care Health Sciences, University of Oxford, Oxford UK

[a] This trial is sponsored by the University of Oxford, Clinical Trials and Research Governance, Joint Research Office, Block 60, Churchill Hospital, Old Road, Headington, Oxford, OX3 7LE, UK.

Keywords: Obesity, diet, weight loss, primary care

For peer review only

ABSTRACT

Introduction

The global prevalence of obesity has risen significantly in recent decades. There is a pressing need to identify effective interventions to treat established obesity that can be delivered at scale. The aim of the DROPLET study is to determine the clinical effectiveness, feasibility and acceptability of referral to a low-energy total diet replacement programme compared with usual weight management interventions in primary care.

Methods and Analysis

The DROPLET trial is a randomised controlled trial comparing a low-energy total diet replacement programme with usual weight management interventions delivered in primary care. Eligible patients will be recruited through primary care registers and randomised to receive a behavioural support programme delivered by their practice nurse or a referral to a commercial provider offering an initial 810 kcal/d low-energy total diet replacement programme for 8 weeks, followed by gradual food reintroduction, along with weekly behavioural support for 24 weeks. The primary outcome is weight change at 12 months. The secondary outcomes are weight change at 3 and 6 months, the proportion of participants achieving 5% and 10% weight loss at 12 months and change in fat mass, HbA1c, LDL cholesterol and systolic and diastolic blood pressure at 12 months. Data will be analysed on the basis of intention to treat. Qualitative interviews on a sub-sample of patients and healthcare providers will assess their experiences of the weight loss programmes and identify factors affecting acceptability and adherence.

Ethics and dissemination

This study has been reviewed and approved by NHS/HRA Research Ethics Committee (Ref: SC/15/0337). The trial findings will be disseminated to academic and health professionals through presentations at meetings and peer reviewed journals and to the public through the media. If the intervention is effective, the results will be communicated to policymakers and commissioners of weight management services.

Trial registration number

ISRCTN75092026

For peer review only

STRENGTHS & LIMITATIONS

- This study is the largest randomised controlled trial to date of a low-energy total diet replacement programme for weight management in routine primary care
- This intervention is based on a model of care where GPs refer patients to a programme delivered in the community by a commercial provider using non-NHS staff, which, if successful, could be readily adopted into practice without the need for specialist training for the primary care workforce
- The primary outcome is weight at one year. Although this is 9 months after the low-energy total diet replacement, epidemiological evidence suggests that any weight lost will continue to be regained beyond one year.
- The intention of obesity treatment programmes is to improve long-term health but this study does not include morbidity or mortality outcomes.
- Longer term follow-up data would be helpful to better estimate the longer health impact and cost effectiveness of the intervention.

INTRODUCTION

The prevalence obesity worldwide has more than doubled since 1980 [1]. According to the latest estimates from the World Health Organization (WHO) more than 1.9 billion adults were overweight, of whom 600 million were obese, representing 39% and 13% of the world's adult population, respectively [2]. Obesity is associated with premature mortality [3] but also substantial morbidity, including significantly increased risks of diabetes, cardiovascular disease and most non-smoking related cancers, as well as physical impairments linked to excess weight such as breathlessness, joint problems and back pain [4]. Collectively this creates a burden of ill-health and reduced quality of life for individuals, additional treatment costs to the NHS and reductions in economic productivity [5].

While high priority must be given to prevent future cases of obesity, in the short term there is a pressing need to identify effective interventions to treat established obesity. Research has shown that even modest reductions in weight can bring significantly reduced risks of disease. For example, in the US Diabetes Prevention Program (DPP) individuals randomised to an intensive lifestyle intervention lost 7kg by the end of the first year. Although some of this weight was regained, the intensive lifestyle group remained 4 kg lighter than the usual care group at four years and this reduced the incidence of diabetes by 58% relative to usual care [6] with benefits persisting to at least 15 year follow-up despite weight regain [7].

Primary care is an important setting for weight management interventions to reduce multi-morbidity. However, although a number of interventions have been shown to be effective in intensive research studies, this success has not always been replicated in routine settings. For

For peer review only

example, there was no significant reduction in weight when a weight loss programme adapted from the DPP was delivered by primary care teams[8]. Our recent review of interventions suitable for use in routine care[9] and a second review, using slightly different inclusion criteria, of interventions specifically delivered in primary care [10] both concluded that behavioural weight management interventions led by primary care practitioners were ineffective. This may relate in part to the complexity of advice needed for successful dietary change and the need for frequent contact to provide support which exceeds the capacity of routine primary care systems. However, although a number of interventions have been shown to be effective in intensive research studies, this success has not always been replicated in routine settings. GP referral to a commercial provider offering group-based support is an effective option for weight management in primary care, and our meta-analysis showed a mean reduction in weight of 2.3 kg over no intervention at one year [9]. However, greater weight losses would be expected to bring greater health gains.

Very low energy diets (VLEDs) have been used for weight loss over many years in specialist settings. A VLED is defined as a diet providing ≤800kcal a day, based on the use of specially formulated products designed as the sole source of nutrition during periods of total diet replacement. When used as directed, these formula products meet 100% of the dietary reference values for vitamins, minerals and trace elements for healthy, weight-stable people and are enriched with high biological-value protein. Although most contain some dietary fibre, a fibre supplement may also be recommended. A recent systematic review and meta-analysis of the available randomised controlled trials showed that behavioural weight management interventions incorporating a VLED led to 3.9 kg greater weight loss at one year compared with intensive specialist-delivered behavioural programmes [11]. However, most of the trials included in this review were small, typically including only 50-100 participants that were treated by obesity specialists and many trials had methodological limitations.

UK guidance from the National Institute for Health and Care Excellence (NICE) recommends that VLEDs may only be used for a maximum of 12 weeks in people who have a clinical need to lose weight rapidly, such as prior to a knee replacement surgery or those seeking fertility services, but recommends against their routine use to manage obesity [12]. Clinical guidance in the USA does not recommend the routine use of VLEDs, but rather suggests that their use "*may* be reasonable in limited circumstances, but only when provided by trained practitioners in a medical care setting where medical monitoring and high intensity lifestyle intervention can be provided" [13]. Nevertheless, there has been growing interest in the potential for routine use of weight loss programmes similar to traditional VLEDs, in so far as they incorporate a period of total diet replacement using specially formulated products as the sole source of nutrition, but where the

For peer review only

energy content is more than 800kcal/day but less than 1200 kcal/day. The NICE guidelines suggest that this type of low-energy diet could be considered for weight management, providing care is taken to ensure they are nutritionally complete [12]. There is one observational report (n = 91) on the use of these low-energy total diet replacement programmes in primary care which found that 64% of participants completed the 810kcal/day dietary programme, defined as either 12 weeks or reaching 20kg weight loss, with a mean weight loss of 16.9 kg (standard deviation (SD) = 6.0 kg). One third of participants starting the programme maintained a weight loss of ≥15 kg at 12 months [14]. A large randomised controlled trial, the DiRECT study, is currently underway to investigate whether this type of low-energy total diet replacement programme can be used to treat type 2 diabetes among people who are also overweight [15]. It will compare the health effects of the current best-available type 2 diabetes care with those achieved through weight management based on a low-energy total diet replacement programme. While this will provide important mechanistic evidence on the links between weight loss and diabetes risk, it will be delivered by NHS staff whereas the present study will test the effectiveness of referral outside the NHS to a commercial provider.

To fill this evidence gap we will conduct a randomised controlled trial to specifically test the effectiveness of a GP referral to a community-based low energy total diet replacement programme for patients who are obese and likely to benefit from weight loss. It will assess the clinical effectiveness of a weight loss intervention by measuring weight loss and the change in biomarkers of cardiovascular risk at 12 months relative to weight loss advice provided by practice nurses. This comparator is intended to represent ‘usual care’, though in practice most patients who are obese are not offered support to lose weight.

The context for this trial follows the established model for GP referral to community group-based weight loss programmes [16]. This uses the generic authority and credibility of health professionals to motivate patients to consider weight management and the specialist knowledge of the commercial provider to guide the intervention and offer frequent contact and behavioural support to the patient. If successful, it will provide another option for weight management that can be offered to patients in primary care, and GPs will be able to guide patients towards the treatment which best fits their circumstances and preferences. This trial will specifically test whether a partnership between GPs and providers will allow for the safe provision of low energy total diet replacement programmes even for patients with multi-morbidity who may gain the greatest benefits from such interventions but who may also need clinical oversight and adjustments to some of their medications as they lose weight. It will provide the opportunity for qualitative research to investigate the perspectives of patients and health care practitioners on this type of treatment.

For peer review only

OBJECTIVE:

The aim of the DROPLET trial is to determine the clinical effectiveness, feasibility and acceptability of referral to a low-energy total diet replacement programme compared with usual weight management interventions in primary care.

For peer review only

METHODS:

Design and Setting

The study will take place in general practices in England. Designed as an individually randomised, two arm and parallel group superiority trial with the primary endpoint as objectively measured changes in body weight from baseline to 12 months. Due to the nature of the intervention it will not be possible to blind participants, clinicians or some of the study team to the treatment allocation after randomisation.

Recruitment

Around 10 general practices will be identified to take part through the clinical research networks. Recruited practices will be asked to conduct a search of their electronic health records in order to identify suitable patients for the DROPLET study. As a result of this search, eligible patients will be sent an invitation letter from their GP as part of a staggered mail out. Patients will be encouraged to call the research team if they are interested in taking part.

GPs may also identify eligible patients during routine consultations. The GP will provide the patient with an invitation letter and suggest that the patient ring the study team. The study team will provide the potential participants with information on what taking part in the study will entail, and an initial assessment of suitability to take part. Those who make contact and self-report meeting the eligibility criteria will be scheduled for a baseline/enrolment appointment.

Inclusion Criteria:

- Participant is willing and able to give informed consent for participation in the study.
- Aged 18 years or above.
- Body Mass Index ≥30 kg/m^2.
- Likely to benefit from weight loss in the GP's opinion.

Exclusion criteria:

- Unable to understand English
- Currently or recently (within 3 months of study entry) attended a weight management programme or currently participating in another weight loss study.
- Had bariatric surgery, or scheduled bariatric surgery.
- Pregnant, breastfeeding, or planning to become pregnant during the course of the study.
- Receiving insulin therapy
- Heart attack or stroke within the last 3 months,
- Heart failure of grade II New York Heart Association and more severe
- Angina, arrhythmia, including atrial fibrillation or prolonged QT syndrome

For peer review only

- Taking MAOI medication
- Taking anticoagulant medication (e.g. warfarin)
- Taking varenicline (smoking cessation medication)
- Chronic renal failure of stage 4 or 5
- Active liver disease (except NAFLD) a past history of hepatoma or within 6 months of onset of acute hepatitis.
- People having active treatment for cancer other than skin cancer treated with curative intent by local treatment only or people taking hormonal or other long-term secondary prevention treatment after initial cancer treatment.
- Active treatment or investigation for possible or confirmed gastric or duodenal ulcer. Maintenance treatment with acid-suppression is not a contra-indication.
- Porphyria
- Scheduled for surgery within 12 months
- A member of household is already enrolled in the study
- Unwilling to provide blood samples
- Patients that the GP judges not able to meet the demands of either treatment programme or measurement schedule. This may include severe medical problems not listed above or severe psychiatric problems including substance misuse that make following the treatment programme or adhering to the protocol unlikely.

Participant Flow

The baseline/eligibility assessment will be scheduled with a practice nurse or health care assistant at their own GP practice, where informed consent for participation in the study will be obtained before eligibility will be formally assessed. After demographic information and all baseline measurements have been collected the participant will be randomised to the allocated treatment group using the online randomisation system. The patients' own GP will be notified by letter of the enrolment and randomisation of their patient, so that it may be documented on their medical record. Participants allocated to the low-energy total diet replacement programme and taking medications for type 2 diabetes, hypertension or high cholesterol will have their medications reviewed by a prescribing member of the clinical care team, usually the GP or trained nurse prescriber. During this medication review the clinician will decide what changes to medications are required at the time the participant commences the low energy total diet replacement programme, with guidance provided by the study team (Supplementary Figure 1). In addition, participants randomised to the low-energy total diet replacement group and who take anti-hypertensive medications will be provided with a home blood pressure monitor and asked to record blood pressure once daily during the weight loss phase (weeks

For peer review only - http://bmjopen.bmj.com/site/about/guidelines.xhtml

1-12). These readings can be used to guide clinicians with any further changes in hypertension medications.

All participants will be invited to attend a 4 week follow-up appointment with the practice nurse. The main purpose of the visit is a clinical review of medication, including any adjustments required. Any changes in medication will be recorded on the concomitant medication log. Participants will be invited to attend further follow-up visits with a member of the trial team at the GP practice at 12 weeks, 6 months and 12 month following randomisation. Participant flow through the study is outlined in **Figure 1**.

Sample size

The total number of participants to be recruited for this study is 270. This is based on a sample size calculation for the primary outcome using equal variance independent samples t-test assuming a difference between groups at 12 months of 4kg with a standard deviation in both groups of 9kg; obtained from a meta-analysis of published studies [11]. The sample size has been inflated by 20%, to account for attrition, and assumes 90% power and two-sided alpha of 5%.

Randomisation

All eligible, consenting participants will be randomised with an allocation ratio of 1:1 to low-energy total diet replacement or usual care programmes using an online program which reveals group allocation as per a computer-generated randomisation list. The randomisation criteria will be validated by an independent statistician. Allocation will be stratified by GP Practice and baseline BMI (≤ 35kg/m2 or > $35kg/m^2$) using stratified block randomisation with randomly varying block sizes of size 2, 4, and 6. The randomisation software ensures full allocation concealment, with the allocation group only revealed to the person performing the randomisation once a study identifier and required stratification details have been entered.

For peer review only

For peer review only - http://bmjopen.bmj.com/site/about/guidelines.xhtml

Interventions

Low-energy total diet replacement

The programme offered to participants randomised to the active intervention will be provided by Cambridge Weight Plan Ltd.™ Northants., United Kingdom.

Following randomisation participants allocated to this group will be referred to a local Cambridge Weight Plan Counsellor who will invite the participant to attend regular appointments for 24 weeks. These appointments consist of motivational support, encouragement, reassurance and problem-solving. All counsellors attend a 1-day in-person training course covering screening for suitability, nutrition, behavioural approaches, and medical monitoring. They must pass and accreditation examination before they are allowed to deliver the programme in the community. Thereafter, they have a yearly training updates, a nominated sponsor (experienced counsellor) and access to an online chat forum for sharing queries. Cambridge Weight Plan has a healthcare professional available for the counsellors to consult for advice on specific medical and nutritional queries. Counsellors delivering the intervention for the purposes of this trial received short trial specific training before being allocated study participants.

During the first 12 weeks the participant will meet with their counsellor weekly. Patients will be asked to follow a programme based on using formula meal replacement products (soups, shakes and bars) and milk comprising 810kcal/day (3389kJ/d). For the first eight weeks patients will be advised to replace *all* their usual foods and drinks with four of the formula products daily, 750ml of skimmed milk, 2.25L of water or other non-calorific drinks and a fibre supplement (total diet replacement stage). During the first two weeks, the formula products will be limited to liquid products (soups and shakes), but from week 3 onwards participants will have the option to include meal replacement bars as part of the formula product allowance. After eight weeks there will be a four week stepwise reduction in the use of formula meal replacement products and a gradual re-introduction of food-based meals. The weight maintenance phase from week 12 to 24 participants attend monthly appointments at 16, 20 and 24 weeks, during this phase participants will consume only one formula product a day, with the remainder of diet provided by food. This weight maintenance phase will include a recommendation to return to the total diet replacement stage for periods of up to 4 weeks if a participant regains 1kg or more than their weight measured at 12 weeks.

All consultations with counsellors and formula products will be provided to participants by their nominated counsellor and will be free of charge for the first 24 weeks, after which the intervention will end. Participants in both groups will be free to choose whether or not to continue with the programme, but at their own cost.

For peer review only

Comparator

The comparator intervention will consist of the usual weight management programme provided by a member of the practice nurse team who has been trained to offer a weight loss programme. The trial will take place only in practices where this is routine care. Participants allocated to the usual care group will not be prevented from attending other weight management groups if they choose to do so, but no NHS referrals to these schemes will be offered during the trial. The practice nurse will give participants a copy of the booklet " So you want to lose weight … for good" [17]. This 47 page booklet provides advice akin to a behavioural weight management programme. The aim is to produce a weight loss goal of 0.5 to 1kg/week. It includes goal setting, advice on portion control and physical activity, other behavioural strategies, and monitoring and feedback on progress. Nurses will be asked to offer a programme for 12 weeks, at a frequency that is usually used in the practice (e.g. weekly or bi-weekly).

Physical activity

We recognise the importance of the role of aerobic and resistance exercise in facilitating weight loss and maintaining lean body mass to facilitate weight loss maintenance.

Participants randomised to the low-energy total diet replacement arm are given appropriate advice based on their previous exercise history, current ability and what is appropriate for their stage weight loss programme. Clinical guidelines in the UK emphasise the importance of advice to increase physical activity and we would expect this to be incorporated into the control 'usual care' intervention.

Outcomes:

Primary outcome

- Change in body weight from baseline to 12 months

Secondary outcomes

- Change in body weight from baseline at 3 and 6 months
- Proportion of participants achieving 5% and 10% weight loss at 12 months
- Change in fat mass between baseline and 12 months
- Change in LDL cholesterol concentrations between baseline and 12 months
- Change in HbA1c between baseline and 12 months
- Change in systolic and diastolic blood pressure between baseline and 12 months

Exploratory outcomes

For peer review only - http://bmjopen.bmj.com/site/about/guidelines.xhtml

- Change in fat mass from baseline to 12 weeks and from baseline to 6 months.
- Change in waist circumference from baseline to 3, 6, and 12 months.
- Change in triglyceride and HDL cholesterol concentrations between baseline and 12 months.
- Change in fasting glucose and insulin concentrations and change in HOMA-IR, HOMA-%S and HOMA-%B between baseline and 12 months.
- Change in systolic and diastolic blood pressure between baseline and 3 months and between baseline and 6 months.
- Change in QRISK between baseline and 12 months.
- Change in the EQ-5D scale between baseline and 12 months
- Change in obesity related quality of life measured with the OWLQOL between baseline, 3, 6, and 12 months.
- Proportion of people continuing their weight loss attempt and following the prescribed programme at 4, 8, and 12 weeks.
- The number of weight control behaviours that participants are using assessed using the OxFAB questionnaire [18] at 3 and 6 months.
- Qualitative interviews with a sub-sample of participants at 6 and 12 months
- Adverse Event reports up to 12 weeks, the end of the weight loss intervention or 6 months for AE's known or presumed to be related to gallstones.

Measurements

Figure 2 provides a summary of the measurements collected.

Socio-demographic characteristics; Participants' will be asked to self-report age, sex, ethnicity

Medical History; Relevant medical history and all concomitant medication will be recorded and checked against the participants' medical record. Participants will also be asked to self-report items required to determine cardiovascular risk score using QRISK2 [19].

Physical measurements; Height will be measured to the nearest 1cm using stadiometers available in the practice. Weight will be recorded to the nearest 0.1kg using an electronic scale (SC240 MA, Tanita Japan) which will also record the proportion of body fat using bioelectrical impedance. Waist circumference will be measured in the horizontal plane at the upper border of the iliac crest at the end of expiration[20] using a fibreglass non-stretch tape measure fitted with a tensioning device (Gulik II Tape Meaure, Fitness Mart USA). Seated blood pressure will be measured in triplicate with 1 min between each measure. All physical measures are performed by assessors trained according to the study manual of procedures.

Fasting blood sample; A fasting venous blood sample will be collected (to be analysed for glucose, insulin, HbA1c, HDL and LDL cholesterol, triglycerides). When baseline/enrolment appointments are

For peer review only

scheduled at times when it may be inappropriate to fast, participants will be asked to arrange for a fasting blood sample to be collected at an alternative appointment within 7 days of the enrolment visit and before the participant commences the allocated weight loss programme.

Questionnaires; Participants will be provided with a questionnaire booklet which they will be asked to complete and return to the trial team in a postage paid envelope provided. The questionnaire booklet contains the following measures:

Obesity specific quality of life (OWLQOL); a weight-specific instrument intended to be used to assess obesity specific symptoms and quality of life, general functional status and well-being, and person-specific preference measurement [21].

Quality of Life; EQ-5D will be used as a standardised validated instrument used for measuring general health status [22].

Programme adherence; Self-reported adherence to the allocated programme and methods participants are using to attempt to lose weight will be recorded by questionnaire.

Programme feedback; will be assessed using several 5-point Likert scales, including whether there is an aim to continue with the programme.

Oxford Food and Activity Behaviours (OxFAB): a questionnaire to assess personal strategies used by individuals for the purposes of weight loss[18].

For peer review only

Retention and withdrawal

We will seek to follow up all participants except those who expressly withdraw from the study. Participants who decide to withdraw from or discontinue the intervention allocated as part of the study will be asked to return for follow-up visits to collect outcome measures. To promote participant retention and complete follow-up participants will be offered a £10 gift card for attending each of the 6 month and 12 month follow-up visits.

Adverse Events

Adverse events are of relevance in this trial because many practitioners feel these programmes are poorly tolerated and unsuitable for routine use in primary care. We will record AEs following Good Clinical Practice (GCP). All serious and non-serious AE's that occur during the first 12 weeks of the study or until the termination of the weight loss programme will be recorded in participants who initiate one of the weight loss interventions. We will also record all AEs that are presumed to be or known to be related to gallstones up to 6 months.

Data management

Data will be recorded in a web-based data capture system (OpenClinica), which is hosted by the Primary Care Clinical Trials Unit of the University Oxford. This system is customised and has an audit trail facility. Ranges and programmed validation checks are implemented in the system in order to aid reliable data entry.

Statistical Analysis

The primary and secondary outcomes will be assessed using an intention-to-treat (ITT) analysis by an independent statistician. Each continuous outcome will be assumed to follow the normal distribution and be analysed by means of a linear mixed effects model, adjusted for outcome at baseline. The model will include fixed effects terms for randomised group, visit, interaction between randomised group and visit, and baseline BMI (for non-weight outcomes only), and random effects to account for repeated measures on the same participant at 3, 6 and 12 months. No adjustment will be made for baseline BMI in the analysis of the weight outcomes due to its strong collinearity with baseline weight. A random effect will also be included for individual practice. An unstructured variance covariance matrix will be specified between repeated measurements on the same individual and the random effects for patient and practice will be assumed to be independent. The adjusted treatment effect together with the 95% confidence interval and p-value will be reported. The analysis will be performed using PROC MIXED in a current version of SAS. The proportion of

For peer review only

participants who lose 5% and 10% of their initial weight at 12 months respectively will be presented and the adjusted difference between two arms and 95% confidence interval will be reported. The binary outcome will be analysed by means of a logistic mixed effects model, adjusting for baseline BMI (fixed effect) and practice (random effect). The number needed to treat (NNT) to achieve 5 or 10% weight loss, defined as the inverse of the absolute difference in proportions, will be reported if the differences between the treatment and control groups are statistically significant. A full statistical analysis plan will be prepared prior to any data analysis.

Qualitative sub-study

The purpose of this study is to examine participants' views of the programmes. In particular, we aim to examine the features that helped or hindered adherence to the programme and participants' views of the behavioural support provided in the respective programmes. We will therefore purposively sample participants based on their responses to the satisfaction questionnaire, reflecting positive, neutral and negative evaluations. Where possible, we will select participants to reflect both genders, socioeconomic status, and ethnic group differences. We anticipate interviewing around 20 participants in the intervention group and 10 in the control group but sampling will continue until saturation is reached, evidenced by no new themes occurring.

We will develop a semi-structured topic guide for the interviews. The interviewer will encourage respondents to discuss their perceptions and experiences freely and in depth. The interview will set the context by asking about previous experience of weight management. Thereafter, we will ask for participants' views on which component parts of their treatment they felt were effective and which they felt were not effective; thoughts about ability to continue to manage their weight when treatment has ended; and their views on medication adjustments where these occurred. The acceptability of the weight management treatment programmes and any preference they initially had for the total diet replacement programme or the usual care programme will be explored.

Data from participants will be collected in a confidential, telephone interview which will be audio recorded. All interviews will be transcribed. To examine saturation, analysis will proceed concurrently with interviewing.

Trial Steering Committee

An independent Trial Steering Committee (TSC) will provide oversight of all matters relating to participant safety and data quality and value to the public. Due to the low risk nature of the DROPLET trial and that it is an open label trial, the TSC also has the role of the Data Monitoring

For peer review only

Committee in addition to their role as the TSC. However, there are no early stopping rules and all AEs are evaluated un-blinded to allocation by the trial management group as well as the TSC. The TSC includes an independent clinician, dietitian, statistician and two patient representatives. The TSC has reviewed the trial protocol, statistical analysis plan and the suitability of the proposed safety data to be collected. No interim analysis is planned for this trial due to the short recruitment period and low risk nature of the two dietary approaches [11]. The trial may be subject to inspection and audit by University of Oxford, under their remit as sponsor, the trial coordinating centre as the Sponsor's delegate and other regulatory bodies.

Ethics and dissemination

The study protocol (Version: 4.0 Date: 5th October 2016) was reviewed and approved by the South Central Oxford B REC Committee (Ref: 157/SC/0337). Any protocol modifications will be sent for review by the research ethics committee and will be amended at the trial registry.

It is planned that results will be disseminated to academic and health professional audiences via presentations at conferences and publication in peer-reviewed journals. Participants will be sent a summary of the trial findings at the time when the main article is published. If the trial shows this intervention is effective, results will be communicated to policymakers and commissioners of weight management services through briefing papers summarising the main findings. We will also provide the results to all participants coincident with publication and disseminate the results to the public through a press release, regardless of what the results show.

Acknowledgements

The low-energy total diet replacement programme including the formula meal replacement products will be provided by Cambridge Weight Plan Ltd, Northants, UK.

Funding and Sponsorship

This research is funded by research grants from Cambridge Weight Plan Ltd and NIHR Collaboration for Leadership in Applied Health Research and Care (CLAHRC) Oxford at Oxford Health NHS Foundation to the University of Oxford. The sponsor of the trial is the University of Oxford.

Contributions

The protocol was initiated and designed by the investigators who have no personal financial relationships with the Cambridge Weight Plan Ltd. Although Cambridge Weight Plan were consulted and commented on the protocol, the final decisions lay with the investigators. There are no

For peer review only - http://bmjopen.bmj.com/site/about/guidelines.xhtml

restrictions on publication of results arising from this study and the contract between the funder and the University ensures that the funding body will have no input into the decisions regarding publication.

SAJ and PA designed the study and secured the funding. NA and ST helped to develop the protocol. NA is the trial manger and AN is the trial statistician.

Competing interests

SAJ and PA have led publicly funded trials in which the weight management intervention was provided free of charge by other commercial companies. They receive no personal financial benefits from these trials or from the companies. NMA, ST, and AN have no competing interests.

Footnotes

As sponsor, the University of Oxford has a specialist insurance policy in place that would operate in the event of any participant suffering harm as a result of their involvement in the research.

Provenance and peer review

Not commissioned; external peer review for ethical approval prior to submission.

Data sharing

For access to the data set, a formal request should be sent to the DROPLET study group. The request will only be considered when the principal results of the study have been published.

For peer review only

References

1. Kelly T, Yang W, Chen C-S, et al. Global burden of obesity in 2005 and projections to 2030. International journal of obesity 2008;**32**(9):1431-37.
2. World Health Organization. Obesity and overweight: fact sheet Geneva: World Health Organization; 2016 [Available from: http://www.who.int/mediacentre/factsheets/fs311/en/.
3. Prospective Studies C, Whitlock G, Lewington S, et al. Body-mass index and cause-specific mortality in 900 000 adults: collaborative analyses of 57 prospective studies. Lancet 2009;**373**(9669):1083-96 doi: 10.1016/S0140-6736(09)60318-4.
4. Guh DP, Zhang W, Bansback N, et al. The incidence of co-morbidities related to obesity and overweight: a systematic review and meta-analysis. BMC Public Health 2009;**9**:88 doi: 10.1186/1471-2458-9-88.
5. Butland B, Jebb SA, McPherson K, et al. Tackling Obesities: Foresight report: Government Office for Science, 2007.
6. Knowler WC, Barrett-Connor E, Fowler SE, et al. Reduction in the incidence of type 2 diabetes with lifestyle intervention or metformin. N Engl J Med 2002;**346**(6):393-403 doi: 10.1056/NEJMoa012512.
7. Diabetes Prevention Program Research G. Long-term effects of lifestyle intervention or metformin on diabetes development and microvascular complications over 15-year follow-up: the Diabetes Prevention Program Outcomes Study. Lancet Diabetes Endocrinol 2015;**3**(11):866-75 doi: 10.1016/S2213-8587(15)00291-0.
8. Vermunt PW, Milder IE, Wielaard F, et al. Lifestyle counseling for type 2 diabetes risk reduction in Dutch primary care: results of the APHRODITE study after 0.5 and 1.5 years. Diabetes Care 2011;**34**(9):1919-25 doi: 10.2337/dc10-2293.
9. Hartmann-Boyce J, Johns DJ, Jebb SA, et al. Behavioural weight management programmes for adults assessed by trials conducted in everyday contexts: systematic review and meta-analysis. Obes Rev 2014;**15**(11):920-32 doi: 10.1111/obr.12220.
10. Booth HP, Prevost TA, Wright AJ, et al. Effectiveness of behavioural weight loss interventions delivered in a primary care setting: a systematic review and meta-analysis. Fam Pract 2014;**31**(6):643-53 doi: 10.1093/fampra/cmu064.
11. Parretti HM, Jebb SA, Johns DJ, et al. Clinical effectiveness of very-low-energy diets in the management of weight loss: a systematic review and meta-analysis of randomized controlled trials. Obes Rev 2016;**17**(3):225-34 doi: 10.1111/obr.12366.
12. (NICE) National Institute for Health and Clinical Excellence Obesity: identification, assesment and management (NICE clinical guideline). London: NICE, 2006.
13. Jensen MD, Ryan DH, Apovian CM, et al. 2013 AHA/ACC/TOS guideline for the management of overweight and obesity in adults: a report of the American College of Cardiology/American Heart Association Task Force on Practice Guidelines and The Obesity Society. Circulation 2014;**129**(25 Suppl 2):S102-38 doi: 10.1161/01.cir.0000437739.71477.ee.
14. Lean M, Brosnahan N, McLoone P, et al. Feasibility and indicative results from a 12-month low-energy liquid diet treatment and maintenance programme for severe obesity. Br J Gen Pract 2013;**63**(607):e115-24 doi: 10.3399/bjgp13X663073.
15. Leslie WS, Ford I, Sattar N, et al. The Diabetes Remission Clinical Trial (DiRECT): protocol for a cluster randomised trial. BMC Fam Pract 2016;**17**:20 doi: 10.1186/s12875-016-0406-2.
16. Ahern AL, Olson AD, Aston LM, et al. Weight Watchers on prescription: an observational study of weight change among adults referred to Weight Watchers by the NHS. BMC Public Health 2011;**11**:434 doi: 10.1186/1471-2458-11-434.
17. British Heart Foundation. *So you want to lose weight... for good*. London: BHF Publications, 2005.
18. Hartmann-Boyce J, Aveyard P, Koshiaris C, et al. Development of tools to study personal weight control strategies: OxFAB taxonomy. Obesity (Silver Spring) 2016;**24**(2):314-20 doi: 10.1002/oby.21341.

For peer review only

19. Hippisley-Cox J, Coupland C, Vinogradova Y, et al. Predicting cardiovascular risk in England and Wales: prospective derivation and validation of QRISK2. BMJ 2008;**336**(7659):1475-82 doi: 10.1136/bmj.39609.449676.25.
20. NHLBI Obesity Education Initiative. The practical guide: Identification, evaluation and treatment of overweight and obesity in adults: National Institutes of Health (NIH Publication Number 00-4084), 2000.
21. Patrick DL, Bushnell DM, Rothman M. Performance of two self-report measures for evaluating obesity and weight loss. Obes Res 2004;**12**(1):48-57 doi: 10.1038/oby.2004.8.
22. EuroQol G. EuroQol--a new facility for the measurement of health-related quality of life. Health Policy 1990;**16**(3):199-208.

For peer review only

Figure 1 : Participant flow through the study

Figure 2: Schedule of measurements

1. Hartmann-Boyce J, Aveyard P, Koshiaris C, et al. Development of tools to study personal weight control strategies: OxFAB taxonomy. Obesity (Silver Spring) 2016;**24**(2):314-20 doi: 10.1002/oby.21341.

For peer review only

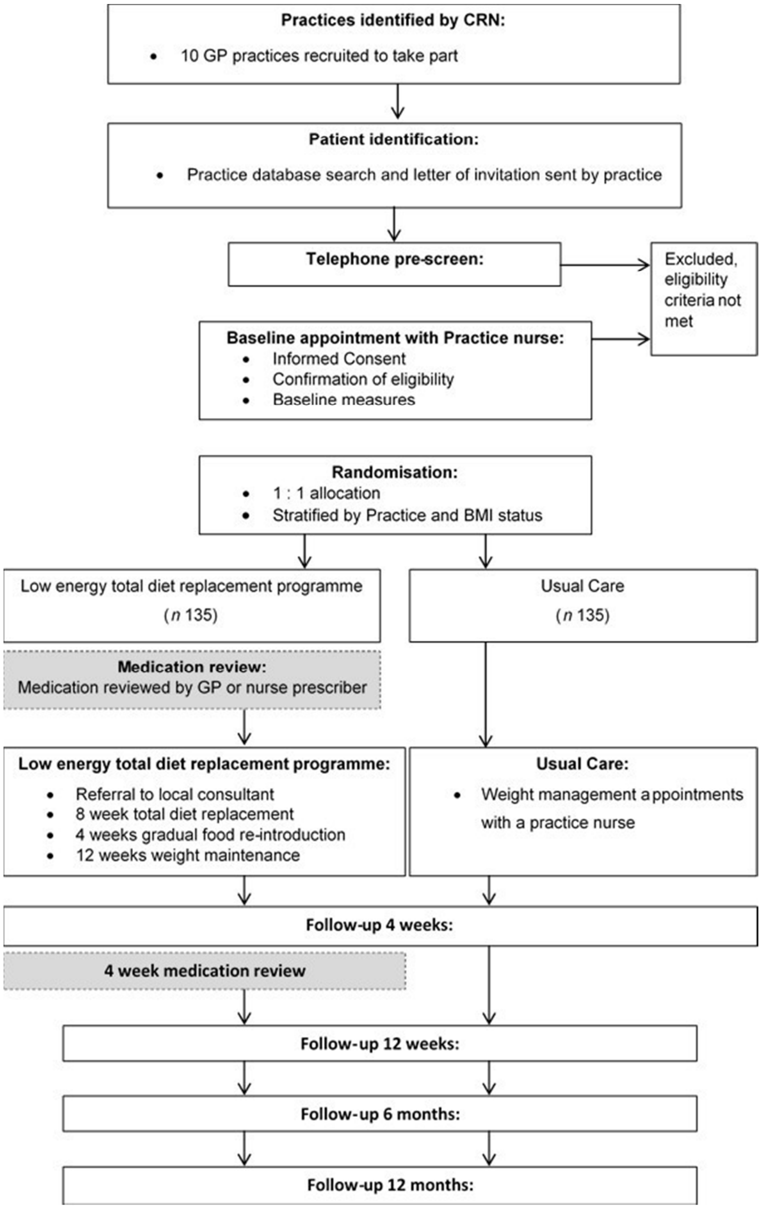

Participant flow through the study

159x252mm (96 x 96 DPI)

	VISIT				
	Enrolment	Follow-up visits			
TIMEPOINT	*Baseline*	*4 weeks*	*12 weeks*	*6 months*	*12 month*
ENROLMENT:					
Informed consent	X				
Eligibility screen	X				
Randomisation	X				
INTERVENTIONS:					
Low energy total diet replacement programme					
Usual Care					
ASSESSMENTS:					
Demographic	X				
Medical History	X				
Concomitant Medication	X	X	X	X	X
Height	X				
Weight	X	X	X	X	X
Body composition	X	X	X	X	X
Waist circumference	X	X	X	X	X
Blood Pressure	X	X	X	X	X
Fasting blood sample	X				X
Medication review	X	X			
LITATIVE INTERVIEWS			X		X
STIONNAIRES:					
Quality of life: (EQ-5D and OWLQOL)	X		X	X	X
Programme adherence		X	X	X	X
Programme feedback		X	X	X	
OXFAB[1]			X	X	

Schedule of measurements

190x275mm (96 x 96 DPI)

Medication changes

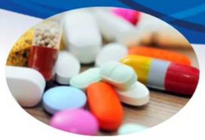

Guidance on making adjustments to your patients' medications

This guidance aims to help you make these medication adjustments, but if in doubt please use your clinical judgement or contact the GP lead for this study.
The adjustments detailed below should be made when commencing the low-energy total diet replacement programme.

TYPE 2 DIABETES

Patient currently takes:	Recommendation
Metformin	HALF daily dose
Sulphonylurea	STOP
Glitazone	STOP
Glinide	STOP
DPP IV inhibitor	STOP
Acarbose	STOP

At the end of the weight loss phase, re-assess patients requirements for oral diabetic therapies using HbA1c measurements or a finger prick blood glucose measurement.

HYPERTENSION

Patient currently takes:	Current dose	Recommendation
Loop Diuretic:		
Furosemide	≤ 40 mg daily	STOP
	80 – 120 mg daily	REDUCE by 40 mg daily
	≥ 120 mg daily	REDUCE by 40 mg daily
Bumetamide	≤ 1 mg daily	STOP
	2-3 mg daily	REDUCE to 1mg daily
	≥ 3 mg daily	REDUCE by 1mg daily
Thiazide Diuretic		STOP
β Blocker	Used for hypertension	STOP
	Other uses	CONTINUE
α Blocker		HALF daily dose
Ca channel blocker		HALF daily dose
ACE inhibitors or ARBs	Used for hypertension	STOP
	Used for heart failure	HALF daily dose

LIPID DRUGS

Patient currently takes:	Recommendation
Fibrates	STOP
Statins	CONTINUE
Ezetimibe	CONTINUE

209x297mm (150 x 150 DPI)

SPIRIT

STANDARD PROTOCOL ITEMS: RECOMMENDATIONS FOR INTERVENTIONAL TRIALS

SPIRIT 2013 Checklist: Recommended items to address in a clinical trial protocol and related documents*

**Section/item**	**Item No**	**Description**	**Addressed on page number**
**Administrative information**			
Title	1	Descriptive title identifying the study design, population, interventions, and, if applicable, trial acronym	P 1
Trial registration	2a	Trial identifier and registry name. If not yet registered, name of intended registry	P2
	2b	All items from the World Health Organization Trial Registration Data Set	
Protocol version	3	Date and version identifier	P15
Funding	4	Sources and types of financial, material, and other support	P15
Roles and responsibilities	5a	Names, affiliations, and roles of protocol contributors	P1
	5b	Name and contact information for the trial sponsor	P1
	5c	Role of study sponsor and funders, if any, in study design; collection, management, analysis, and interpretation of data; writing of the report; and the decision to submit the report for publication, including whether they will have ultimate authority over any of these activities	P15

For peer review only - http://bmjopen.bmj.com/site/about/guidelines.xhtml

	5d	Composition, roles, and responsibilities of the coordinating centre, steering committee, endpoint adjudication committee, data management team, and other individuals or groups overseeing the trial, if applicable (see Item 21a for data monitoring committee)	P14
**Introduction**			
Background and rationale	6a	Description of research question and justification for undertaking the trial, including summary of relevant studies (published and unpublished) examining benefits and harms for each intervention	P4-6
	6b	Explanation for choice of comparators	P10-11
Objectives	7	Specific objectives or hypotheses	P6
Trial design	8	Description of trial design including type of trial (eg, parallel group, crossover, factorial, single group), allocation ratio, and framework (eg, superiority, equivalence, noninferiority, exploratory)	P7
**Methods: Participants, interventions, and outcomes**			
Study setting	9	Description of study settings (eg, community clinic, academic hospital) and list of countries where data will be collected. Reference to where list of study sites can be obtained	P7
Eligibility criteria	10	Inclusion and exclusion criteria for participants. If applicable, eligibility criteria for study centres and individuals who will perform the interventions (eg, surgeons, psychotherapists)	P7-8
Interventions	11a	Interventions for each group with sufficient detail to allow replication, including how and when they will be administered	P10-11
	11b	Criteria for discontinuing or modifying allocated interventions for a given trial participant (eg, drug dose change in response to harms, participant request, or improving/worsening disease)	P13
	11c	Strategies to improve adherence to intervention protocols, and any procedures for monitoring adherence (eg, drug tablet return, laboratory tests)	P13
	11d	Relevant concomitant care and interventions that are permitted or prohibited during the trial	N/A

For peer review only - http://bmjopen.bmj.com/site/about/guidelines.xhtml

Outcomes	12	Primary, secondary, and other outcomes, including the specific measurement variable (eg, systolic blood pressure), analysis metric (eg, change from baseline, final value, time to event), method of aggregation (eg, median, proportion), and time point for each outcome. Explanation of the clinical relevance of chosen efficacy and harm outcomes is strongly recommended	P11
Participant timeline	13	Time schedule of enrolment, interventions (including any run-ins and washouts), assessments, and visits for participants. A schematic diagram is highly recommended (see Figure)	Figure 2
Sample size	14	Estimated number of participants needed to achieve study objectives and how it was determined, including clinical and statistical assumptions supporting any sample size calculations	P9
Recruitment	15	Strategies for achieving adequate participant enrolment to reach target sample size	P7
**Methods: Assignment of interventions (for controlled trials)**			
Allocation:			
Sequence generation	16a	Method of generating the allocation sequence (eg, computer-generated random numbers), and list of any factors for stratification. To reduce predictability of a random sequence, details of any planned restriction (eg, blocking) should be provided in a separate document that is unavailable to those who enrol participants or assign interventions	P9
Allocation concealment mechanism	16b	Mechanism of implementing the allocation sequence (eg, central telephone; sequentially numbered, opaque, sealed envelopes), describing any steps to conceal the sequence until interventions are assigned	P9
Implementation	16c	Who will generate the allocation sequence, who will enrol participants, and who will assign participants to interventions	P9
Blinding (masking)	17a	Who will be blinded after assignment to interventions (eg, trial participants, care providers, outcome assessors, data analysts), and how	P9
	17b	If blinded, circumstances under which unblinding is permissible, and procedure for revealing a participant's allocated intervention during the trial	N/A
**Methods: Data collection, management, and analysis**			

Data collection methods	18a	Plans for assessment and collection of outcome, baseline, and other trial data, including any related processes to promote data quality (eg, duplicate measurements, training of assessors) and a description of study instruments (eg, questionnaires, laboratory tests) along with their reliability and validity, if known. Reference to where data collection forms can be found, if not in the protocol	P12
	18b	Plans to promote participant retention and complete follow-up, including list of any outcome data to be collected for participants who discontinue or deviate from intervention protocols	P13
Data management	19	Plans for data entry, coding, security, and storage, including any related processes to promote data quality (eg, double data entry; range checks for data values). Reference to where details of data management procedures can be found, if not in the protocol	P13
Statistical methods	20a	Statistical methods for analysing primary and secondary outcomes. Reference to where other details of the statistical analysis plan can be found, if not in the protocol	P13/14
	20b	Methods for any additional analyses (eg, subgroup and adjusted analyses)	P13/14
	20c	Definition of analysis population relating to protocol non-adherence (eg, as randomised analysis), and any statistical methods to handle missing data (eg, multiple imputation)	P13
**Methods: Monitoring**			
Data monitoring	21a	Composition of data monitoring committee (DMC); summary of its role and reporting structure; statement of whether it is independent from the sponsor and competing interests; and reference to where further details about its charter can be found, if not in the protocol. Alternatively, an explanation of why a DMC is not needed	P14/15
	21b	Description of any interim analyses and stopping guidelines, including who will have access to these interim results and make the final decision to terminate the trial	N/A
Harms	22	Plans for collecting, assessing, reporting, and managing solicited and spontaneously reported adverse events and other unintended effects of trial interventions or trial conduct	P13
Auditing	23	Frequency and procedures for auditing trial conduct, if any, and whether the process will be independent from investigators and the sponsor	P15

For peer review only - http://bmjopen.bmj.com/site/about/guidelines.xhtml

**Ethics and dissemination**			
Research ethics approval	24	Plans for seeking research ethics committee/institutional review board (REC/IRB) approval	P15
Protocol amendments	25	Plans for communicating important protocol modifications (eg, changes to eligibility criteria, outcomes, analyses) to relevant parties (eg, investigators, REC/IRBs, trial participants, trial registries, journals, regulators)	P15
Consent or assent	26a	Who will obtain informed consent or assent from potential trial participants or authorised surrogates, and how (see Item 32)	P8
	26b	Additional consent provisions for collection and use of participant data and biological specimens in ancillary studies, if applicable	P8
Confidentiality	27	How personal information about potential and enrolled participants will be collected, shared, and maintained in order to protect confidentiality before, during, and after the trial	______________
Declaration of interests	28	Financial and other competing interests for principal investigators for the overall trial and each study site	P16
Access to data	29	Statement of who will have access to the final trial dataset, and disclosure of contractual agreements that limit such access for investigators	_P16
Ancillary and post-trial care	30	Provisions, if any, for ancillary and post-trial care, and for compensation to those who suffer harm from trial participation	_N/A
Dissemination policy	31a	Plans for investigators and sponsor to communicate trial results to participants, healthcare professionals, the public, and other relevant groups (eg, via publication, reporting in results databases, or other data sharing arrangements), including any publication restrictions	P15
	31b	Authorship eligibility guidelines and any intended use of professional writers	P15-16
	31c	Plans, if any, for granting public access to the full protocol, participant-level dataset, and statistical code	P16
**Appendices**			

For peer review only - http://bmjopen.bmj.com/site/about/guidelines.xhtml

Informed consent materials	32	Model consent form and other related documentation given to participants and authorised surrogates	________
Biological specimens	33	Plans for collection, laboratory evaluation, and storage of biological specimens for genetic or molecular analysis in the current trial and for future use in ancillary studies, if applicable	________

*It is strongly recommended that this checklist be read in conjunction with the SPIRIT 2013 Explanation & Elaboration for important clarification on the items. Amendments to the protocol should be tracked and dated. The SPIRIT checklist is copyrighted by the SPIRIT Group under the Creative Commons "Attribution-NonCommercial-NoDerivs 3.0 Unported" license.

For peer review only

BMJ Open

Doctor referral of overweight people to a low-energy treatment (DROPLET) in primary care using total diet replacement products: a protocol for a randomised controlled trial

Journal:	*BMJ Open*
Manuscript ID	bmjopen-2017-016709.R2
Article Type:	Protocol
Date Submitted by the Author:	13-Jun-2017
Complete List of Authors:	Jebb, Susan; University of Oxford, Nuffield Department of Primary Care Health Sciences<br>Astbury, Nerys; University of Oxford, Nuffield Department of Primary Care Health Sciences<br>Tearne, Sarah; University of Oxford, Nuffield Department of Primary Care Health Sciences<br>Nickless, Alecia; University of Oxford, Nuffield Department of Primary Care Health Sciences<br>Aveyard, Paul; University of Oxford, Nuffield Department of Primary Care Health Sciences
**Primary Subject Heading**:	General practice / Family practice
Secondary Subject Heading:	Nutrition and metabolism
Keywords:	Obesity, Diet, Weight loss, PRIMARY CARE

SCHOLARONE™
Manuscripts

Doctor referral of overweight people to a low-energy treatment (DROPLET) in primary care using total diet replacement products: a protocol for a randomised controlled trial [a]

Susan A Jebb [1]

Nerys M Astbury [1]

Sarah Tearne [1]

Alecia Nickless [1]

Paul Aveyard [1]

*Corresponding Author: Nuffield Department of Primary Care Health Sciences, Radcliffe Primary Care Building, Woodstock Road, Oxford, OX2 6GG, email: nerys.astbury@phc.ox.ac.uk, tel: 01865 617871

[1] Nuffield Department of Primary Care Health Sciences, University of Oxford, Oxford UK

[a] This trial is sponsored by the University of Oxford, Clinical Trials and Research Governance, Joint Research Office, Block 60, Churchill Hospital, Old Road, Headington, Oxford, OX3 7LE, UK.

Keywords: Obesity, diet, weight loss, primary care

For peer review only

ABSTRACT

Introduction

The global prevalence of obesity has risen significantly in recent decades. There is a pressing need to identify effective interventions to treat established obesity that can be delivered at scale. The aim of the DROPLET study is to determine the clinical effectiveness, feasibility and acceptability of referral to a low-energy total diet replacement programme compared with usual weight management interventions in primary care.

Methods and Analysis

The DROPLET trial is a randomised controlled trial comparing a low-energy total diet replacement programme with usual weight management interventions delivered in primary care. Eligible patients will be recruited through primary care registers and randomised to receive a behavioural support programme delivered by their practice nurse or a referral to a commercial provider offering an initial 810 kcal/d low-energy total diet replacement programme for 8 weeks, followed by gradual food reintroduction, along with weekly behavioural support for 24 weeks. The primary outcome is weight change at 12 months. The secondary outcomes are weight change at 3 and 6 months, the proportion of participants achieving 5% and 10% weight loss at 12 months and change in fat mass, HbA1c, LDL cholesterol and systolic and diastolic blood pressure at 12 months. Data will be analysed on the basis of intention to treat. Qualitative interviews on a sub-sample of patients and healthcare providers will assess their experiences of the weight loss programmes and identify factors affecting acceptability and adherence.

Ethics and dissemination

This study has been reviewed and approved by NHS/HRA Research Ethics Committee (Ref: SC/15/0337). The trial findings will be disseminated to academic and health professionals through presentations at meetings and peer reviewed journals and to the public through the media. If the intervention is effective, the results will be communicated to policymakers and commissioners of weight management services.

Trial registration number

ISRCTN75092026

For peer review only

STRENGTHS & LIMITATIONS

- This study is the largest randomised controlled trial to date of a low-energy total diet replacement programme for weight management in routine primary care
- This intervention is based on a model of care where GPs refer patients to a programme delivered in the community by a commercial provider using non-NHS staff, which, if successful, could be readily adopted into practice without the need for specialist training for the primary care workforce
- The primary outcome is weight at one year. Although this is 9 months after the low-energy total diet replacement, epidemiological evidence suggests that any weight lost will continue to be regained beyond one year.
- The intention of obesity treatment programmes is to improve long-term health but this study does not include morbidity or mortality outcomes.
- Longer term follow-up data would be helpful to better estimate the longer health impact and cost effectiveness of the intervention.

INTRODUCTION

The prevalence obesity worldwide has more than doubled since 1980 [1]. According to the latest estimates from the World Health Organization (WHO) more than 1.9 billion adults were overweight, of whom 600 million were obese, representing 39% and 13% of the world's adult population, respectively [2]. Obesity is associated with premature mortality [3] but also substantial morbidity, including significantly increased risks of diabetes, cardiovascular disease and most non-smoking related cancers, as well as physical impairments linked to excess weight such as breathlessness, joint problems and back pain [4]. Collectively this creates a burden of ill-health and reduced quality of life for individuals, additional treatment costs to the NHS and reductions in economic productivity [5].

While high priority must be given to prevent future cases of obesity, in the short term there is a pressing need to identify effective interventions to treat established obesity. Research has shown that even modest reductions in weight can bring significantly reduced risks of disease. For example, in the US Diabetes Prevention Program (DPP) individuals randomised to an intensive lifestyle intervention lost 7kg by the end of the first year. Although some of this weight was regained, the intensive lifestyle group remained 4 kg lighter than the usual care group at four years and this reduced the incidence of diabetes by 58% relative to usual care [6] with benefits persisting to at least 15 year follow-up despite weight regain [7].

Primary care is an important setting for weight management interventions to reduce multi-morbidity. However, although a number of interventions have been shown to be effective in intensive research studies, this success has not always been replicated in routine settings. For

For peer review only

example, there was no significant reduction in weight when a weight loss programme adapted from the DPP was delivered by primary care teams[8]. Our recent review of interventions suitable for use in routine care[9] and a second review, using slightly different inclusion criteria, of interventions specifically delivered in primary care [10] both concluded that behavioural weight management interventions led by primary care practitioners were ineffective. This may relate in part to the complexity of advice needed for successful dietary change and the need for frequent contact to provide support which exceeds the capacity of routine primary care systems. However, although a number of interventions have been shown to be effective in intensive research studies, this success has not always been replicated in routine settings. GP referral to a commercial provider offering group-based support is an effective option for weight management in primary care, and our meta-analysis showed a mean reduction in weight of 2.3 kg over no intervention at one year [9]. However, greater weight losses would be expected to bring greater health gains.

Very low energy diets (VLEDs) have been used for weight loss over many years in specialist settings. A VLED is defined as a diet providing ≤800kcal a day, based on the use of specially formulated products designed as the sole source of nutrition during periods of total diet replacement. When used as directed, these formula products meet 100% of the dietary reference values for vitamins, minerals and trace elements for healthy, weight-stable people and are enriched with high biological-value protein. Although most contain some dietary fibre, a fibre supplement may also be recommended. A recent systematic review and meta-analysis of the available randomised controlled trials showed that behavioural weight management interventions incorporating a VLED led to 3.9 kg greater weight loss at one year compared with intensive specialist-delivered behavioural programmes [11]. However, most of the trials included in this review were small, typically including only 50-100 participants that were treated by obesity specialists and many trials had methodological limitations.

UK guidance from the National Institute for Health and Care Excellence (NICE) recommends that VLEDs may only be used for a maximum of 12 weeks in people who have a clinical need to lose weight rapidly, such as prior to a knee replacement surgery or those seeking fertility services, but recommends against their routine use to manage obesity [12]. Clinical guidance in the USA does not recommend the routine use of VLEDs, but rather suggests that their use "*may* be reasonable in limited circumstances, but only when provided by trained practitioners in a medical care setting where medical monitoring and high intensity lifestyle intervention can be provided" [13]. Nevertheless, there has been growing interest in the potential for routine use of weight loss programmes similar to traditional VLEDs, in so far as they incorporate a period of total diet replacement using specially formulated products as the sole source of nutrition, but where the

For peer review only - http://bmjopen.bmj.com/site/about/guidelines.xhtml

energy content is more than 800kcal/day but less than 1200 kcal/day. The NICE guidelines suggest that this type of low-energy diet could be considered for weight management, providing care is taken to ensure they are nutritionally complete [12]. There is one observational report (n = 91) on the use of these low-energy total diet replacement programmes in primary care which found that 64% of participants completed the 810kcal/day dietary programme, defined as either 12 weeks or reaching 20kg weight loss, with a mean weight loss of 16.9 kg (standard deviation (SD) = 6.0 kg). One third of participants starting the programme maintained a weight loss of ≥15 kg at 12 months [14]. A large randomised controlled trial, the DiRECT study, is currently underway to investigate whether this type of low-energy total diet replacement programme can be used to treat type 2 diabetes among people who are also overweight [15]. It will compare the health effects of the current best-available type 2 diabetes care with those achieved through weight management based on a low-energy total diet replacement programme. While this will provide important mechanistic evidence on the links between weight loss and diabetes risk, it will be delivered by NHS staff whereas the present study will test the effectiveness of referral outside the NHS to a commercial provider.

To fill this evidence gap we will conduct a randomised controlled trial to specifically test the effectiveness of a GP referral to a community-based low energy total diet replacement programme for patients who are obese and likely to benefit from weight loss. It will assess the clinical effectiveness of a weight loss intervention by measuring weight loss and the change in biomarkers of cardiovascular risk at 12 months relative to weight loss advice provided by practice nurses. This comparator is intended to represent 'usual care', though in practice most patients who are obese are not offered support to lose weight.

The context for this trial follows the established model for GP referral to community group-based weight loss programmes [16]. This uses the generic authority and credibility of health professionals to motivate patients to consider weight management and the specialist knowledge of the commercial provider to guide the intervention and offer frequent contact and behavioural support to the patient. If successful, it will provide another option for weight management that can be offered to patients in primary care, and GPs will be able to guide patients towards the treatment which best fits their circumstances and preferences. This trial will specifically test whether a partnership between GPs and providers will allow for the safe provision of low energy total diet replacement programmes even for patients with multi-morbidity who may gain the greatest benefits from such interventions but who may also need clinical oversight and adjustments to some of their medications as they lose weight. It will provide the opportunity for qualitative research to investigate the perspectives of patients and health care practitioners on this type of treatment.

For peer review only

OBJECTIVE:

The aim of the DROPLET trial is to determine the clinical effectiveness, feasibility and acceptability of referral to a low-energy total diet replacement programme compared with usual weight management interventions in primary care.

For peer review only

METHODS:

Design and Setting

The study will take place in general practices in England. Designed as an individually randomised, two arm and parallel group superiority trial with the primary endpoint as objectively measured changes in body weight from baseline to 12 months. Due to the nature of the intervention it will not be possible to blind participants, clinicians or some of the study team to the treatment allocation after randomisation.

Recruitment

Around 10 general practices will be identified to take part through the clinical research networks. Recruited practices will be asked to conduct a search of their electronic health records in order to identify suitable patients for the DROPLET study. As a result of this search, eligible patients will be sent an invitation letter from their GP as part of a staggered mail out. Patients will be encouraged to call the research team if they are interested in taking part.

GPs may also identify eligible patients during routine consultations. The GP will provide the patient with an invitation letter and suggest that the patient ring the study team. The study team will provide the potential participants with information on what taking part in the study will entail, and an initial assessment of suitability to take part. Those who make contact and self-report meeting the eligibility criteria will be scheduled for a baseline/enrolment appointment.

Inclusion Criteria:

- Participant is willing and able to give informed consent for participation in the study.
- Aged 18 years or above.
- Body Mass Index ≥30 kg/m^2.
- Likely to benefit from weight loss in the GP's opinion.

Exclusion criteria:

- Unable to understand English
- Currently or recently (within 3 months of study entry) attended a weight management programme or currently participating in another weight loss study.
- Had bariatric surgery, or scheduled bariatric surgery.
- Pregnant, breastfeeding, or planning to become pregnant during the course of the study.
- Receiving insulin therapy
- Heart attack or stroke within the last 3 months,
- Heart failure of grade II New York Heart Association and more severe
- Angina, arrhythmia, including atrial fibrillation or prolonged QT syndrome

For peer review only

- Taking MAOI medication
- Taking anticoagulant medication (e.g. warfarin)
- Taking varenicline (smoking cessation medication)
- Chronic renal failure of stage 4 or 5
- Active liver disease (except NAFLD) a past history of hepatoma or within 6 months of onset of acute hepatitis.
- People having active treatment for cancer other than skin cancer treated with curative intent by local treatment only or people taking hormonal or other long-term secondary prevention treatment after initial cancer treatment.
- Active treatment or investigation for possible or confirmed gastric or duodenal ulcer. Maintenance treatment with acid-suppression is not a contra-indication.
- Porphyria
- Scheduled for surgery within 12 months
- A member of household is already enrolled in the study
- Unwilling to provide blood samples
- Patients that the GP judges not able to meet the demands of either treatment programme or measurement schedule. This may include severe medical problems not listed above or severe psychiatric problems including substance misuse that make following the treatment programme or adhering to the protocol unlikely.

Participant Flow

The baseline/eligibility assessment will be scheduled with a practice nurse or health care assistant at their own GP practice, where informed consent for participation in the study will be obtained before eligibility will be formally assessed. After demographic information and all baseline measurements have been collected the participant will be randomised to the allocated treatment group using the online randomisation system. The patients' own GP will be notified by letter of the enrolment and randomisation of their patient, so that it may be documented on their medical record. Participants allocated to the low-energy total diet replacement programme and taking medications for type 2 diabetes, hypertension or high cholesterol will have their medications reviewed by a prescribing member of the clinical care team, usually the GP or trained nurse prescriber. During this medication review the clinician will decide what changes to medications are required at the time the participant commences the low energy total diet replacement programme, with guidance provided by the study team (Supplementary Figure 1). In addition, participants randomised to the low-energy total diet replacement group and who take anti-hypertensive medications will be provided with a home blood pressure monitor and asked to record blood pressure once daily during the weight loss phase (weeks

For peer review only - http://bmjopen.bmj.com/site/about/guidelines.xhtml

1-12). These readings can be used to guide clinicians with any further changes in hypertension medications.

All participants will be invited to attend a 4 week follow-up appointment with the practice nurse. The main purpose of the visit is a clinical review of medication, including any adjustments required. Any changes in medication will be recorded on the concomitant medication log. Participants will be invited to attend further follow-up visits with a member of the trial team at the GP practice at 12 weeks, 6 months and 12 month following randomisation. Participant flow through the study is outlined in **Figure 1**.

Sample size

The total number of participants to be recruited for this study is 270. This is based on a sample size calculation for the primary outcome using equal variance independent samples t-test assuming a difference between groups at 12 months of 4kg with a standard deviation in both groups of 9kg; obtained from a meta-analysis of published studies [11]. The sample size has been inflated by 20%, to account for attrition, and assumes 90% power and two-sided alpha of 5%.

Randomisation

All eligible, consenting participants will be randomised with an allocation ratio of 1:1 to low-energy total diet replacement or usual care programmes using an online program which reveals group allocation as per a computer-generated randomisation list. The randomisation criteria will be validated by an independent statistician. Allocation will be stratified by GP Practice and baseline BMI (≤ 35kg/m2 or > 35kg/m^2) using stratified block randomisation with randomly varying block sizes of size 2, 4, and 6. The randomisation software ensures full allocation concealment, with the allocation group only revealed to the person performing the randomisation once a study identifier and required stratification details have been entered.

For peer review only

Interventions

Low-energy total diet replacement

The programme offered to participants randomised to the active intervention will be provided by Cambridge Weight Plan Ltd.™ Northants., United Kingdom.

Following randomisation participants allocated to this group will be referred to a local Cambridge Weight Plan Counsellor who will invite the participant to attend regular appointments for 24 weeks. These appointments consist of motivational support, encouragement, reassurance and problem-solving. All counsellors attend a 1-day in-person training course covering screening for suitability, nutrition, behavioural approaches, and medical monitoring. They must pass and accreditation examination before they are allowed to deliver the programme in the community. Thereafter, they have a yearly training updates, a nominated sponsor (experienced counsellor) and access to an online chat forum for sharing queries. Cambridge Weight Plan has a healthcare professional available for the counsellors to consult for advice on specific medical and nutritional queries. Counsellors delivering the intervention for the purposes of this trial received short trial specific training before being allocated study participants.

During the first 12 weeks the participant will meet with their counsellor weekly. Patients will be asked to follow a programme based on using formula meal replacement products (soups, shakes and bars) and milk comprising 810kcal/day (3389kJ/d). For the first eight weeks patients will be advised to replace *all* their usual foods and drinks with four of the formula products daily, 750ml of skimmed milk, 2.25L of water or other non-calorific drinks and a fibre supplement (total diet replacement stage). During the first two weeks, the formula products will be limited to liquid products (soups and shakes), but from week 3 onwards participants will have the option to include meal replacement bars as part of the formula product allowance. After eight weeks there will be a four week stepwise reduction in the use of formula meal replacement products and a gradual re-introduction of food-based meals. The weight maintenance phase from week 12 to 24 participants attend monthly appointments at 16, 20 and 24 weeks, during this phase participants will consume only one formula product a day, with the remainder of diet provided by food. This weight maintenance phase will include a recommendation to return to the total diet replacement stage for periods of up to 4 weeks if a participant regains 1kg or more than their weight measured at 12 weeks.

All consultations with counsellors and formula products will be provided to participants by their nominated counsellor and will be free of charge for the first 24 weeks, after which the intervention will end. Participants in both groups will be free to choose whether or not to continue with the programme, but at their own cost.

For peer review only

Comparator

The comparator intervention will consist of the usual weight management programme provided by a member of the practice nurse team who has been trained to offer a weight loss programme. The trial will take place only in practices where this is routine care. Participants allocated to the usual care group will not be prevented from attending other weight management groups if they choose to do so, but no NHS referrals to these schemes will be offered during the trial. The practice nurse will give participants a copy of the booklet " So you want to lose weight … for good" [17]. This 47 page booklet provides advice akin to a behavioural weight management programme. The aim is to produce a weight loss goal of 0.5 to 1kg/week. It includes goal setting, advice on portion control and physical activity, other behavioural strategies, and monitoring and feedback on progress. Nurses will be asked to offer a programme for 12 weeks, at a frequency that is usually used in the practice (e.g. weekly or bi-weekly).

Physical activity

We recognise the importance of the role of aerobic and resistance exercise in facilitating weight loss and maintaining lean body mass to facilitate weight loss maintenance.

Participants randomised to the low-energy total diet replacement arm are given appropriate advice based on their previous exercise history, current ability and what is appropriate for their stage weight loss programme. Clinical guidelines in the UK emphasise the importance of advice to increase physical activity and we would expect this to be incorporated into the control 'usual care' intervention.

Outcomes:

Primary outcome

- Change in body weight from baseline to 12 months

Secondary outcomes

- Change in body weight from baseline at 3 and 6 months
- Proportion of participants achieving 5% and 10% weight loss at 12 months
- Change in fat mass between baseline and 12 months
- Change in LDL cholesterol concentrations between baseline and 12 months
- Change in HbA1c between baseline and 12 months
- Change in systolic and diastolic blood pressure between baseline and 12 months

Exploratory outcomes

For peer review only

- Change in fat mass from baseline to 12 weeks and from baseline to 6 months.
- Change in waist circumference from baseline to 3, 6, and 12 months.
- Change in triglyceride and HDL cholesterol concentrations between baseline and 12 months.
- Change in fasting glucose and insulin concentrations and change in HOMA-IR, HOMA-%S and HOMA-%B between baseline and 12 months.
- Change in systolic and diastolic blood pressure between baseline and 3 months and between baseline and 6 months.
- Change in QRISK between baseline and 12 months.
- Change in the EQ-5D scale between baseline and 12 months
- Change in obesity related quality of life measured with the OWLQOL between baseline, 3, 6, and 12 months.
- Proportion of people continuing their weight loss attempt and following the prescribed programme at 4, 8, and 12 weeks.
- The number of weight control behaviours that participants are using assessed using the OxFAB questionnaire [18] at 3 and 6 months.
- Qualitative interviews with a sub-sample of participants at 6 and 12 months
- Adverse Event reports up to 12 weeks, the end of the weight loss intervention or 6 months for AE's known or presumed to be related to gallstones.

Measurements

Figure 2 provides a summary of the measurements collected.

Socio-demographic characteristics; Participants' will be asked to self-report age, sex, ethnicity

Medical History; Relevant medical history and all concomitant medication will be recorded and checked against the participants' medical record. Participants will also be asked to self-report items required to determine cardiovascular risk score using QRISK2 [19].

Physical measurements; Height will be measured to the nearest 1cm using stadiometers available in the practice. Weight will be recorded to the nearest 0.1kg using an electronic scale (SC240 MA, Tanita Japan) which will also record the proportion of body fat using bioelectrical impedance. Waist circumference will be measured in the horizontal plane at the upper border of the iliac crest at the end of expiration[20] using a fibreglass non-stretch tape measure fitted with a tensioning device (Gulik II Tape Meaure, Fitness Mart USA). Seated blood pressure will be measured in triplicate with 1 min between each measure. All physical measures are performed by assessors trained according to the study manual of procedures.

Fasting blood sample; A fasting venous blood sample will be collected (to be analysed for glucose, insulin, HbA1c, HDL and LDL cholesterol, triglycerides). When baseline/enrolment appointments are

For peer review only

scheduled at times when it may be inappropriate to fast, participants will be asked to arrange for a fasting blood sample to be collected at an alternative appointment within 7 days of the enrolment visit and before the participant commences the allocated weight loss programme.

Questionnaires; Participants will be provided with a questionnaire booklet which they will be asked to complete and return to the trial team in a postage paid envelope provided. The questionnaire booklet contains the following measures:

Obesity specific quality of life (OWLQOL); a weight-specific instrument intended to be used to assess obesity specific symptoms and quality of life, general functional status and well-being, and person-specific preference measurement [21].

Quality of Life; EQ-5D will be used as a standardised validated instrument used for measuring general health status [22].

Programme adherence; Self-reported adherence to the allocated programme and methods participants are using to attempt to lose weight will be recorded by questionnaire.

Programme feedback; will be assessed using several 5-point Likert scales, including whether there is an aim to continue with the programme.

Oxford Food and Activity Behaviours (OxFAB): a questionnaire to assess personal strategies used by individuals for the purposes of weight loss[18].

For peer review only

Retention and withdrawal

We will seek to follow up all participants except those who expressly withdraw from the study. Participants who decide to withdraw from or discontinue the intervention allocated as part of the study will be asked to return for follow-up visits to collect outcome measures. To promote participant retention and complete follow-up participants will be offered a £10 gift card for attending each of the 6 month and 12 month follow-up visits.

Adverse Events

Adverse events are of relevance in this trial because many practitioners feel these programmes are poorly tolerated and unsuitable for routine use in primary care. We will record AEs following Good Clinical Practice (GCP). All serious and non-serious AE's that occur during the first 12 weeks of the study or until the termination of the weight loss programme will be recorded in participants who initiate one of the weight loss interventions. We will also record all AEs that are presumed to be or known to be related to gallstones up to 6 months.

Data management

Data will be recorded in a web-based data capture system (OpenClinica), which is hosted by the Primary Care Clinical Trials Unit of the University Oxford. This system is customised and has an audit trail facility. Ranges and programmed validation checks are implemented in the system in order to aid reliable data entry.

Statistical Analysis

The primary and secondary outcomes will be assessed using an intention-to-treat (ITT) analysis by an independent statistician. Each continuous outcome will be assumed to follow the normal distribution and be analysed by means of a linear mixed effects model, adjusted for outcome at baseline. The model will include fixed effects terms for randomised group, visit, interaction between randomised group and visit, and baseline BMI (for non-weight outcomes only), and random effects to account for repeated measures on the same participant at 3, 6 and 12 months. No adjustment will be made for baseline BMI in the analysis of the weight outcomes due to its strong collinearity with baseline weight. A random effect will also be included for individual practice. An unstructured variance covariance matrix will be specified between repeated measurements on the same individual and the random effects for patient and practice will be assumed to be independent. The adjusted treatment effect together with the 95% confidence interval and p-value will be reported. The analysis will be performed using PROC MIXED in a current version of SAS. The proportion of

For peer review only

participants who lose 5% and 10% of their initial weight at 12 months respectively will be presented and the adjusted difference between two arms and 95% confidence interval will be reported. The binary outcome will be analysed by means of a logistic mixed effects model, adjusting for baseline BMI (fixed effect) and practice (random effect). The number needed to treat (NNT) to achieve 5 or 10% weight loss, defined as the inverse of the absolute difference in proportions, will be reported if the differences between the treatment and control groups are statistically significant. A full statistical analysis plan will be prepared prior to any data analysis.

Qualitative sub-study

The purpose of this study is to examine participants' views of the programmes. In particular, we aim to examine the features that helped or hindered adherence to the programme and participants' views of the behavioural support provided in the respective programmes. We will therefore purposively sample participants based on their responses to the satisfaction questionnaire, reflecting positive, neutral and negative evaluations. Where possible, we will select participants to reflect both genders, socioeconomic status, and ethnic group differences. We anticipate interviewing around 20 participants in the intervention group and 10 in the control group but sampling will continue until saturation is reached, evidenced by no new themes occurring.

We will develop a semi-structured topic guide for the interviews. The interviewer will encourage respondents to discuss their perceptions and experiences freely and in depth. The interview will set the context by asking about previous experience of weight management. Thereafter, we will ask for participants' views on which component parts of their treatment they felt were effective and which they felt were not effective; thoughts about ability to continue to manage their weight when treatment has ended; and their views on medication adjustments where these occurred. The acceptability of the weight management treatment programmes and any preference they initially had for the total diet replacement programme or the usual care programme will be explored.

Data from participants will be collected in a confidential, telephone interview which will be audio recorded. All interviews will be transcribed. To examine saturation, analysis will proceed concurrently with interviewing.

Trial Steering Committee

An independent Trial Steering Committee (TSC) will provide oversight of all matters relating to participant safety and data quality and value to the public. Due to the low risk nature of the DROPLET trial and that it is an open label trial, the TSC also has the role of the Data Monitoring

For peer review only

Committee in addition to their role as the TSC. However, there are no early stopping rules and all AEs are evaluated un-blinded to allocation by the trial management group as well as the TSC. The TSC includes an independent clinician, dietitian, statistician and two patient representatives. The TSC has reviewed the trial protocol, statistical analysis plan and the suitability of the proposed safety data to be collected. No interim analysis is planned for this trial due to the short recruitment period and low risk nature of the two dietary approaches [11]. The trial may be subject to inspection and audit by University of Oxford, under their remit as sponsor, the trial coordinating centre as the Sponsor's delegate and other regulatory bodies.

Ethics and dissemination

The study protocol (Version: 4.0 Date: 5th October 2016) was reviewed and approved by the South Central Oxford B REC Committee (Ref: 157/SC/0337). Any protocol modifications will be sent for review by the research ethics committee and will be amended at the trial registry.

It is planned that results will be disseminated to academic and health professional audiences via presentations at conferences and publication in peer-reviewed journals. Participants will be sent a summary of the trial findings at the time when the main article is published. If the trial shows this intervention is effective, results will be communicated to policymakers and commissioners of weight management services through briefing papers summarising the main findings. We will also provide the results to all participants coincident with publication and disseminate the results to the public through a press release, regardless of what the results show.

Acknowledgements

The low-energy total diet replacement programme including the formula meal replacement products will be provided by Cambridge Weight Plan Ltd, Northants, UK.

Funding and Sponsorship

This research is funded by research grants from Cambridge Weight Plan Ltd and NIHR Collaboration for Leadership in Applied Health Research and Care (CLAHRC) Oxford at Oxford Health NHS Foundation to the University of Oxford. The sponsor of the trial is the University of Oxford.

Contributions

The protocol was initiated and designed by the investigators who have no personal financial relationships with the Cambridge Weight Plan Ltd. Although Cambridge Weight Plan were consulted and commented on the protocol, the final decisions lay with the investigators. There are no

For peer review only - http://bmjopen.bmj.com/site/about/guidelines.xhtml

restrictions on publication of results arising from this study and the contract between the funder and the University ensures that the funding body will have no input into the decisions regarding publication.

SAJ and PA designed the study and secured the funding. NA and ST helped to develop the protocol. NA is the trial manger and AN is the trial statistician.

Competing interests

SAJ and PA have led publicly funded trials in which the weight management intervention was provided free of charge by other commercial companies. They receive no personal financial benefits from these trials or from the companies. NMA, ST, and AN have no competing interests. Cambridge Weight Plan Ltd, as the funder of this trial is also the manufacturer of the nutritional products used in the trial, and provided the products used in the trial free of charge to the participants.

Footnotes

As sponsor, the University of Oxford has a specialist insurance policy in place that would operate in the event of any participant suffering harm as a result of their involvement in the research.

Provenance and peer review

Not commissioned; external peer review for ethical approval prior to submission.

Data sharing

For access to the data set, a formal request should be sent to the DROPLET study group. The request will only be considered when the principal results of the study have been published.

For peer review only

References

1. Kelly T, Yang W, Chen C-S, et al. Global burden of obesity in 2005 and projections to 2030. International journal of obesity 2008;**32**(9):1431-37.
2. World Health Organization. Obesity and overweight: fact sheet Geneva: World Health Organization; 2016 [Available from: http://www.who.int/mediacentre/factsheets/fs311/en/.
3. Prospective Studies C, Whitlock G, Lewington S, et al. Body-mass index and cause-specific mortality in 900 000 adults: collaborative analyses of 57 prospective studies. Lancet 2009;**373**(9669):1083-96 doi: 10.1016/S0140-6736(09)60318-4.
4. Guh DP, Zhang W, Bansback N, et al. The incidence of co-morbidities related to obesity and overweight: a systematic review and meta-analysis. BMC Public Health 2009;**9**:88 doi: 10.1186/1471-2458-9-88.
5. Butland B, Jebb SA, McPherson K, et al. Tackling Obesities: Foresight report: Government Office for Science, 2007.
6. Knowler WC, Barrett-Connor E, Fowler SE, et al. Reduction in the incidence of type 2 diabetes with lifestyle intervention or metformin. N Engl J Med 2002;**346**(6):393-403 doi: 10.1056/NEJMoa012512.
7. Diabetes Prevention Program Research G. Long-term effects of lifestyle intervention or metformin on diabetes development and microvascular complications over 15-year follow-up: the Diabetes Prevention Program Outcomes Study. Lancet Diabetes Endocrinol 2015;**3**(11):866-75 doi: 10.1016/S2213-8587(15)00291-0.
8. Vermunt PW, Milder IE, Wielaard F, et al. Lifestyle counseling for type 2 diabetes risk reduction in Dutch primary care: results of the APHRODITE study after 0.5 and 1.5 years. Diabetes Care 2011;**34**(9):1919-25 doi: 10.2337/dc10-2293.
9. Hartmann-Boyce J, Johns DJ, Jebb SA, et al. Behavioural weight management programmes for adults assessed by trials conducted in everyday contexts: systematic review and meta-analysis. Obes Rev 2014;**15**(11):920-32 doi: 10.1111/obr.12220.
10. Booth HP, Prevost TA, Wright AJ, et al. Effectiveness of behavioural weight loss interventions delivered in a primary care setting: a systematic review and meta-analysis. Fam Pract 2014;**31**(6):643-53 doi: 10.1093/fampra/cmu064.
11. Parretti HM, Jebb SA, Johns DJ, et al. Clinical effectiveness of very-low-energy diets in the management of weight loss: a systematic review and meta-analysis of randomized controlled trials. Obes Rev 2016;**17**(3):225-34 doi: 10.1111/obr.12366.
12. (NICE) National Institute for Health and Clinical Excellence Obesity: identification, assesment and management (NICE clinical guideline). London: NICE, 2006.
13. Jensen MD, Ryan DH, Apovian CM, et al. 2013 AHA/ACC/TOS guideline for the management of overweight and obesity in adults: a report of the American College of Cardiology/American Heart Association Task Force on Practice Guidelines and The Obesity Society. Circulation 2014;**129**(25 Suppl 2):S102-38 doi: 10.1161/01.cir.0000437739.71477.ee.
14. Lean M, Brosnahan N, McLoone P, et al. Feasibility and indicative results from a 12-month low-energy liquid diet treatment and maintenance programme for severe obesity. Br J Gen Pract 2013;**63**(607):e115-24 doi: 10.3399/bjgp13X663073.
15. Leslie WS, Ford I, Sattar N, et al. The Diabetes Remission Clinical Trial (DiRECT): protocol for a cluster randomised trial. BMC Fam Pract 2016;**17**:20 doi: 10.1186/s12875-016-0406-2.
16. Ahern AL, Olson AD, Aston LM, et al. Weight Watchers on prescription: an observational study of weight change among adults referred to Weight Watchers by the NHS. BMC Public Health 2011;**11**:434 doi: 10.1186/1471-2458-11-434.
17. British Heart Foundation. *So you want to lose weight... for good*. London: BHF Publications, 2005.
18. Hartmann-Boyce J, Aveyard P, Koshiaris C, et al. Development of tools to study personal weight control strategies: OxFAB taxonomy. Obesity (Silver Spring) 2016;**24**(2):314-20 doi: 10.1002/oby.21341.

For peer review only - http://bmjopen.bmj.com/site/about/guidelines.xhtml

19. Hippisley-Cox J, Coupland C, Vinogradova Y, et al. Predicting cardiovascular risk in England and Wales: prospective derivation and validation of QRISK2. BMJ 2008;**336**(7659):1475-82 doi: 10.1136/bmj.39609.449676.25.
20. NHLBI Obesity Education Initiative. The practical guide: Identification, evaluation and treatment of overweight and obesity in adults: National Institutes of Health (NIH Publication Number 00-4084), 2000.
21. Patrick DL, Bushnell DM, Rothman M. Performance of two self-report measures for evaluating obesity and weight loss. Obes Res 2004;**12**(1):48-57 doi: 10.1038/oby.2004.8.
22. EuroQol G. EuroQol--a new facility for the measurement of health-related quality of life. Health Policy 1990;**16**(3):199-208.

For peer review only

Figure 1 : Participant flow through the study

Figure 2: Schedule of measurements

1. Hartmann-Boyce J, Aveyard P, Koshiaris C, et al. Development of tools to study personal weight control strategies: OxFAB taxonomy. Obesity (Silver Spring) 2016;**24**(2):314-20 doi: 10.1002/oby.21341.

For peer review only

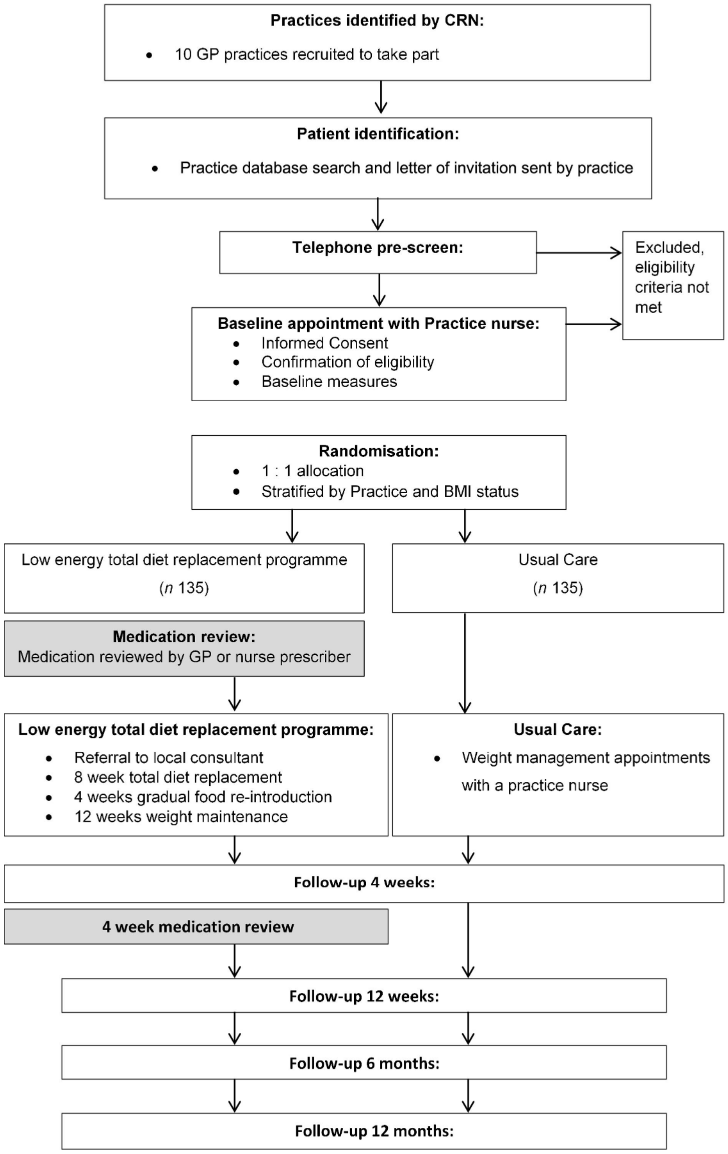

Figure 1: Participant flow through the study

159x265mm (300 x 300 DPI)

	VISIT				
	Enrolment	Follow-up visits			
**TIMEPOINT**	*Baseline*	*4 weeks*	*12 weeks*	*6 months*	*12 months*
**ENROLMENT:**					
Informed consent	X				
Eligibility screen	X				
Randomisation	X				
**INTERVENTIONS:**					
Low energy total diet replacement programme					
Usual Care					
**ASSESSMENTS:**					
Demographic	X				
Medical History	X				
Concomitant Medication	X	X	X	X	X
Height	X				
Weight	X	X	X	X	X
Body composition	X	X	X	X	X
Waist circumference	X	X	X	X	X
Blood Pressure	X	X	X	X	X
Fasting blood sample	X				X
Medication review	X	X			
**QUALITATIVE INTERVIEWS**			X		X
**QUESTIONNAIRES:**					
Quality of life: (EQ-5D and OWLQOL)	X		X	X	X
Programme adherence		X	X	X	X
Programme feedback		X	X	X	
OXFAB[1]			X	X	

Figure 2: Schedule of measurements

176x215mm (300 x 300 DPI)

Medication changes

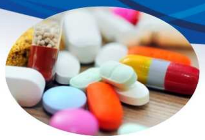

Guidance on making adjustments to your patients' medications

This guidance aimS to help you make these medication adjustments, but please use your clinical judgement or contact the GP lead for this study.

TYPE 2 DIABETES	
**Patient currently takes:**	**Recommendation**
Metformin	HALF daily dose
Sulphonylurea	STOP
Glitazone	STOP
Glinide	STOP
DPP IV inhibitor	STOP
Acarbose	STOP

At the end of the weight loss phase, re-assess patients requirements for oral diabetic therapies using

HYPERTENSION		
**Patient currently takes:**	**Current dose**	**Recommendation**
Loop Diuretic:		
Furosemide	≤ 40 mg daily	STOP
	80 – 120 mg daily	REDUCE by 40 mg daily
	≥ 120 mg daily	REDUCE by 40 mg daily
Bumetamide	≤ 1 mg daily	STOP
	2-3 mg daily	REDUCE to 1mg daily
	≥ 3 mg daily	REDUCE by 1mg daily
Thiazide Diuretic		STOP
▯ Blocker	Used for hypertension	STOP
	Other uses	CONTINUE
▯ Blocker		HALF daily dose
Ca channel blocker		HALF daily dose
ACE inhibitors or ARBs	Used for hypertension	STOP
	Used for heart failure	HALF daily dose

LIPID DRUGS	
**Patient currently takes:**	**Recommendation**
Fibrates	STOP
Statins	CONTINUE
Ezetimibe	CONTINUE

209x297mm (300 x 300 DPI)

STANDARD PROTOCOL ITEMS: RECOMMENDATIONS FOR INTERVENTIONAL TRIALS

SPIRIT 2013 Checklist: Recommended items to address in a clinical trial protocol and related documents*

Section/item	Item No	Description	Addressed on page number
**Administrative information**			
Title	1	Descriptive title identifying the study design, population, interventions, and, if applicable, trial acronym	P 1
Trial registration	2a	Trial identifier and registry name. If not yet registered, name of intended registry	P2
	2b	All items from the World Health Organization Trial Registration Data Set	
Protocol version	3	Date and version identifier	P15
Funding	4	Sources and types of financial, material, and other support	P15
Roles and responsibilities	5a	Names, affiliations, and roles of protocol contributors	P1
	5b	Name and contact information for the trial sponsor	P1
	5c	Role of study sponsor and funders, if any, in study design; collection, management, analysis, and interpretation of data; writing of the report; and the decision to submit the report for publication, including whether they will have ultimate authority over any of these activities	P15

For peer review only - http://bmjopen.bmj.com/site/about/guidelines.xhtml

	5d	Composition, roles, and responsibilities of the coordinating centre, steering committee, endpoint adjudication committee, data management team, and other individuals or groups overseeing the trial, if applicable (see Item 21a for data monitoring committee)	P14
**Introduction**			
Background and rationale	6a	Description of research question and justification for undertaking the trial, including summary of relevant studies (published and unpublished) examining benefits and harms for each intervention	P4-6
	6b	Explanation for choice of comparators	P10-11
Objectives	7	Specific objectives or hypotheses	P6
Trial design	8	Description of trial design including type of trial (eg, parallel group, crossover, factorial, single group), allocation ratio, and framework (eg, superiority, equivalence, noninferiority, exploratory)	P7
**Methods: Participants, interventions, and outcomes**			
Study setting	9	Description of study settings (eg, community clinic, academic hospital) and list of countries where data will be collected. Reference to where list of study sites can be obtained	P7
Eligibility criteria	10	Inclusion and exclusion criteria for participants. If applicable, eligibility criteria for study centres and individuals who will perform the interventions (eg, surgeons, psychotherapists)	P7-8
Interventions	11a	Interventions for each group with sufficient detail to allow replication, including how and when they will be administered	P10-11
	11b	Criteria for discontinuing or modifying allocated interventions for a given trial participant (eg, drug dose change in response to harms, participant request, or improving/worsening disease)	P13
	11c	Strategies to improve adherence to intervention protocols, and any procedures for monitoring adherence (eg, drug tablet return, laboratory tests)	P13
	11d	Relevant concomitant care and interventions that are permitted or prohibited during the trial	N/A

For peer review only - http://bmjopen.bmj.com/site/about/guidelines.xhtml

Outcomes	12	Primary, secondary, and other outcomes, including the specific measurement variable (eg, systolic blood pressure), analysis metric (eg, change from baseline, final value, time to event), method of aggregation (eg, median, proportion), and time point for each outcome. Explanation of the clinical relevance of chosen efficacy and harm outcomes is strongly recommended	P11
Participant timeline	13	Time schedule of enrolment, interventions (including any run-ins and washouts), assessments, and visits for participants. A schematic diagram is highly recommended (see Figure)	Figure 2
Sample size	14	Estimated number of participants needed to achieve study objectives and how it was determined, including clinical and statistical assumptions supporting any sample size calculations	P9
Recruitment	15	Strategies for achieving adequate participant enrolment to reach target sample size	P7
**Methods: Assignment of interventions (for controlled trials)**			
Allocation:			
Sequence generation	16a	Method of generating the allocation sequence (eg, computer-generated random numbers), and list of any factors for stratification. To reduce predictability of a random sequence, details of any planned restriction (eg, blocking) should be provided in a separate document that is unavailable to those who enrol participants or assign interventions	P9
Allocation concealment mechanism	16b	Mechanism of implementing the allocation sequence (eg, central telephone; sequentially numbered, opaque, sealed envelopes), describing any steps to conceal the sequence until interventions are assigned	P9
Implementation	16c	Who will generate the allocation sequence, who will enrol participants, and who will assign participants to interventions	P9
Blinding (masking)	17a	Who will be blinded after assignment to interventions (eg, trial participants, care providers, outcome assessors, data analysts), and how	P9
	17b	If blinded, circumstances under which unblinding is permissible, and procedure for revealing a participant's allocated intervention during the trial	N/A
**Methods: Data collection, management, and analysis**			

Data collection methods	18a	Plans for assessment and collection of outcome, baseline, and other trial data, including any related processes to promote data quality (eg, duplicate measurements, training of assessors) and a description of study instruments (eg, questionnaires, laboratory tests) along with their reliability and validity, if known. Reference to where data collection forms can be found, if not in the protocol	P12
	18b	Plans to promote participant retention and complete follow-up, including list of any outcome data to be collected for participants who discontinue or deviate from intervention protocols	P13
Data management	19	Plans for data entry, coding, security, and storage, including any related processes to promote data quality (eg, double data entry; range checks for data values). Reference to where details of data management procedures can be found, if not in the protocol	P13
Statistical methods	20a	Statistical methods for analysing primary and secondary outcomes. Reference to where other details of the statistical analysis plan can be found, if not in the protocol	P13/14
	20b	Methods for any additional analyses (eg, subgroup and adjusted analyses)	P13/14
	20c	Definition of analysis population relating to protocol non-adherence (eg, as randomised analysis), and any statistical methods to handle missing data (eg, multiple imputation)	P13
**Methods: Monitoring**			
Data monitoring	21a	Composition of data monitoring committee (DMC); summary of its role and reporting structure; statement of whether it is independent from the sponsor and competing interests; and reference to where further details about its charter can be found, if not in the protocol. Alternatively, an explanation of why a DMC is not needed	P14/15
	21b	Description of any interim analyses and stopping guidelines, including who will have access to these interim results and make the final decision to terminate the trial	N/A
Harms	22	Plans for collecting, assessing, reporting, and managing solicited and spontaneously reported adverse events and other unintended effects of trial interventions or trial conduct	P13
Auditing	23	Frequency and procedures for auditing trial conduct, if any, and whether the process will be independent from investigators and the sponsor	P15

For peer review only - http://bmjopen.bmj.com/site/about/guidelines.xhtml

**Ethics and dissemination**			
Research ethics approval	24	Plans for seeking research ethics committee/institutional review board (REC/IRB) approval	P15
Protocol amendments	25	Plans for communicating important protocol modifications (eg, changes to eligibility criteria, outcomes, analyses) to relevant parties (eg, investigators, REC/IRBs, trial participants, trial registries, journals, regulators)	P15
Consent or assent	26a	Who will obtain informed consent or assent from potential trial participants or authorised surrogates, and how (see Item 32)	P8
	26b	Additional consent provisions for collection and use of participant data and biological specimens in ancillary studies, if applicable	P8
Confidentiality	27	How personal information about potential and enrolled participants will be collected, shared, and maintained in order to protect confidentiality before, during, and after the trial	______________
Declaration of interests	28	Financial and other competing interests for principal investigators for the overall trial and each study site	P16
Access to data	29	Statement of who will have access to the final trial dataset, and disclosure of contractual agreements that limit such access for investigators	_P16
Ancillary and post-trial care	30	Provisions, if any, for ancillary and post-trial care, and for compensation to those who suffer harm from trial participation	_N/A
Dissemination policy	31a	Plans for investigators and sponsor to communicate trial results to participants, healthcare professionals, the public, and other relevant groups (eg, via publication, reporting in results databases, or other data sharing arrangements), including any publication restrictions	P15
	31b	Authorship eligibility guidelines and any intended use of professional writers	P15-16
	31c	Plans, if any, for granting public access to the full protocol, participant-level dataset, and statistical code	P16
**Appendices**			

For peer review only - http://bmjopen.bmj.com/site/about/guidelines.xhtml

Informed consent materials	32	Model consent form and other related documentation given to participants and authorised surrogates	______________
Biological specimens	33	Plans for collection, laboratory evaluation, and storage of biological specimens for genetic or molecular analysis in the current trial and for future use in ancillary studies, if applicable	______________

*It is strongly recommended that this checklist be read in conjunction with the SPIRIT 2013 Explanation & Elaboration for important clarification on the items. Amendments to the protocol should be tracked and dated. The SPIRIT checklist is copyrighted by the SPIRIT Group under the Creative Commons "Attribution-NonCommercial-NoDerivs 3.0 Unported" license.

For peer review only
